# Accuracy of ARGOS Locations of Pinnipeds at-Sea Estimated Using Fastloc GPS

**DOI:** 10.1371/journal.pone.0008677

**Published:** 2010-01-15

**Authors:** Daniel P. Costa, Patrick W. Robinson, John P. Y. Arnould, Autumn-Lynn Harrison, Samantha E. Simmons, Jason L. Hassrick, Andrew J. Hoskins, Stephen P. Kirkman, Herman Oosthuizen, Stella Villegas-Amtmann, Daniel E. Crocker

**Affiliations:** 1 Department of Ecology and Evolutionary Biology, University of California Santa Cruz, Santa Cruz, California, United States of America; 2 School of Life and Environmental Sciences, Deakin University, Burwood, Victoria, Australia; 3 Marine and Coastal Management, Department of Environmental Affairs, Rogge Bay, South Africa; 4 Animals Demography Unit, Department of Zoology, University of Cape Town, Rondebosch, South Africa; 5 Department of Biology, Sonoma State University, Rohnert Park, California, United States of America; Institut Pluridisciplinaire Hubert Curien, France

## Abstract

**Background:**

ARGOS satellite telemetry is one of the most widely used methods to track the movements of free-ranging marine and terrestrial animals and is fundamental to studies of foraging ecology, migratory behavior and habitat-use. ARGOS location estimates do not include complete error estimations, and for many marine organisms, the most commonly acquired locations (Location Class 0, A, B, or Z) are provided with no declared error estimate.

**Methodology/Principal Findings:**

We compared the accuracy of ARGOS locations to those obtained using Fastloc GPS from the same electronic tags on five species of pinnipeds: 9 California sea lions (*Zalophus californianus*), 4 Galapagos sea lions (*Zalophus wollebaeki*), 6 Cape fur seals (*Arctocephalus pusillus pusillus*), 3 Australian fur seals (*A. p. doriferus*) and 5 northern elephant seals (*Mirounga angustirostris*). These species encompass a range of marine habitats (highly pelagic *vs* coastal), diving behaviors (mean dive durations 2–21 min) and range of latitudes (equator to temperate). A total of 7,318 ARGOS positions and 27,046 GPS positions were collected. Of these, 1,105 ARGOS positions were obtained within five minutes of a GPS position and were used for comparison. The 68^th^ percentile ARGOS location errors as measured in this study were LC-3 0.49 km, LC-2 1.01 km, LC-1 1.20 km, LC-0 4.18 km, LC-A 6.19 km, LC-B 10.28 km.

**Conclusions/Significance:**

The ARGOS errors measured here are greater than those provided by ARGOS, but within the range of other studies. The error was non-normally distributed with each LC highly right-skewed. Locations of species that make short duration dives and spend extended periods on the surface (sea lions and fur seals) had less error than species like elephant seals that spend more time underwater and have shorter surface intervals. Supplemental data (S1) are provided allowing the creation of density distributions that can be used in a variety of filtering algorithms to improve the quality of ARGOS tracking data.

## Introduction

The ability to track the movements of animals is fundamental to understanding animal foraging ecology, migratory behavior, habitat use and general life history parameters [1], [2], [3]. Electronic tracking technology revolutionized the study of animal ecology and is now relied upon as the predominant means of estimating animal movement [4]. A number of electronic tracking methods are available including VHF telemetry, light-level geolocation, ARGOS satellite telemetry and, most recently, GPS tracking [5], [6], [7], [8], [9], [10], [11]. Of these, ARGOS telemetry is one of the most widely used in marine studies [12] and has the significant advantage that the data are acquired remotely in real time via satellite anywhere on the planet (www.ARGOS-system.org). ARGOS satellite telemetry has expanded our understanding of the fine scale movements of marine birds, [13], [14], [15], sea turtles [16], [17], sharks [18], [19] and marine mammals [7], [11], [12], [20], [21].

The ARGOS system consists of modules attached to low-orbiting weather satellites of the National Oceanic and Atmospheric Administration (NOAA). Modules record satellite tag transmissions and later download these data back to Earth for processing by Service ARGOS (Toulouse France, or Landover MD). Tag geoposition is calculated from the Doppler shift of the transmitted radio frequency and requires a minimum of three successive transmissions during a single satellite pass. Locations are based on 7 levels of accuracy: location class (LC) LC-3 with a stated error of less than 150 m, LC-2 with error of 150–350 m, LC-1 with error of 350–1000 m, LC-0 with error greater than 1000 m, and LC-A, LC-B and LC-Z that do not include error estimates.

Unfortunately, the least accurate positions are the most common for many marine animal tracking studies [22]. For example, 89% of ARGOS location estimates from a recent northern elephant seal track (Costa et al. *unpublished data*) were of LC 0, A, B, or Z and, thus, had no declared error estimate. Furthermore, the errors for the remaining location classes, as provided by ARGOS-CLS, are 68^th^ percentile predictions (separately for latitude and longitude) rather than full error distributions making it difficult to incorporate into animal movement models.

Spatial error is problematic for many applications, such as determination of area-restricted search, behavioral mode (i.e. foraging, searching, migrating, etc) of far-ranging species, or even simple home-range analyses for individuals exhibiting range-residency [23], [24], [25], [26], [27]. If unknown, spatial error can also mask accurate descriptions of true location as well as derived-behavioral states in a scale-dependent fashion. That is, smaller scale patterns will show increased degradation with the addition of ARGOS error. The temporal resolution of tracking data can have similarly detrimental effects on its behavioral interpretation. However, ARGOS transmitters are often set to a fixed transmission rate, so that the temporal resolution of the resulting track is solely dependent on animal behavior. Fortunately, a variety of interpolation techniques are available to mitigate variations in temporal resolution [7], [24], [27], [28]. In contrast, spatial error is propagated both as a result of animal behavior (surfacing behavior, travel speed etc.) and technical limitations [26]. To date, spatial error of ARGOS positions has not been measured in an empirically robust manner for free-ranging marine animals. The first attempts to estimate ARGOS location error in marine animals were made by comparing the known position of an ARGOS tag prior to deployment or while the animal remained at a known location [29]. However, such estimates failed to account for animal behavior at sea. Factors contributing to location errors when animals are at sea include attenuated or missed transmissions due to rapid surfacing behavior, limited total time available for transmissions, and the commonly overlooked impact of temperature-induced frequency instability [30], [31], [32], [33]. Vincent and others [34] attached ARGOS transmitters to captive gray seals, *Halichoerus grypus*, housed in outdoor tanks to simulate the natural environment and diving behavior of free-ranging seals. They compared the known position of the tank to the locations provided by the ARGOS transmitters and found a lower uplink rate and a shift toward poorer location qualities (0, A, and B) and discovered that ARGOS errors were non-normally distributed. Using acoustic telemetry, White and Sjoberg (2002) followed three free-ranging gray seals on which they had deployed ARGOS transmitters, and took comparative GPS positions from their vessel. They reported location accuracies for LC-0 LC-A, LC-B those not reported by Service ARGOS. Recently Hazel (2009) using a combination of Fastloc GPS and acoustic devices with three green sea turtles, *Chelonia midas*, found ARGOS errors greater than those reported by ARGOS [35]. However, the tracking devices were housed in a float tethered to the turtles making comparisons to studies where the tracking devices are directly attached to the animal (on the head or back) problematic. While these studies provide useful insights into ARGOS error, they do not provide a robust assessment of ARGOS location error for free ranging marine animals under the conditions most existing data have been collected.

Until recently GPS technology could not be used on diving animals because of the time required for the antenna to be at the surface and clear of wave splash to obtain a satellite fix and maintain the almanac. The recent development of Fastloc GPS technology has made it possible to simultaneously collect GPS and ARGOS locations from freely-ranging marine animals (Fastloc GPS hardware developed by Wildtrack Telemetry Systems Ltd, Leeds, United Kingdom). Standard navigational GPS units require many seconds or even minutes of exposure to GPS satellites to calculate positions and the onboard calculations consume considerable power. In contrast, Fastloc GPS can obtain GPS satellite information in less than 100 ms and can transmit the location information within the narrow bandwidth confines of the ARGOS system. The Fastloc uses a novel intermediate solution that couples brief satellite reception with limited onboard processing to reduce the memory required to store or transmit the location. This system captures the GPS satellite signals, identifies the observed satellites, calculates their pseudo-ranges without the ephemeris or satellite almanac and then transmits the pseudo-ranges via ARGOS. While only a portion of the locations can be transmitted via ARGOS, all of them are archived and therefore can be retrieved as long as the tag is recovered. Final locations are post-processed from the pseudo-ranges once the data are received using archived GPS constellation orbitography archives maintained by NASA (http://cddis.gsfc.nasa.gov).

Here, we assess the accuracy of ARGOS locations via comparisons to Fastloc GPS locations obtained from the same electronic tags on five species of pinnipeds. These species cover a range of marine habitats (highly pelagic vs coastal), diving behaviors (mean dive durations 2–21 min) and range of latitudes (equator to temperate) and a wide range of locations across the globe (South Africa, Eastern North Pacific Ocean, Southern California Bight, southeast Australia and the Galapagos Islands).

## Methods

### Ethics Statement

The animal use protocol for this research was reviewed and approved by the University of California at Santa Cruz Institutional Animal Care and Use Committee and followed the guidelines established by the Canadian Council on Animal Care and the ethics committee of the Society of Marine Mammalogy. Research was carried out under the following research permits: northern elephant seals and California sea lions, U.S.A. National Marine Fisheries Service permits #87-1743 and 87-1851; Galapagos sea lions, Parque Nacional Galapagos #084/06PNG; Australian fur seals, Department of Natural Resources and Environment research permits 978/003, RP-97-112 amd 10000187; and Cape fur seals permit issued by the Ministry of Marine and Coastal Management to H. Oosthuizen.

### Data Collection

To assess *in-situ* error of ARGOS positions, we attached ARGOS-linked Fastloc GPS tags (manufactured by Wildlife Computers, Sirtrack Ltd., or the Sea Mammal Research Unit, SMRU Ltd) to individuals representing five species of pinnipeds. Nine California sea lions (*Zalophus californianus*) were captured on San Nicolas Island, California, USA in November 2007 and recaptured in January 2008. Four Galapagos sea lions (*Zalophus wollebaeki*) were captured on Caamaño Islet, Galapagos, Ecuador during August 2006 and recaptured two weeks later. Six Cape fur seals (*Arctocephalus pusillus pusillus*) were captured at Kleinsee seal colony, South Africa in June 2007 and recaptured in July 2007. Three Australian fur seals (*A. p. doriferus*) were captured at Kanowna Island, northern Bass Strait, Australia in June 2006 and were recaptured two weeks later. Five northern elephant seals (*Mirounga angustirostris*) were captured at Año Nuevo State Park, San Mateo County, California, USA in February 2008 and recaptured in May 2008. We used standard capture, sedation, and instrument attachment protocols [11], [36], [37]; all individuals were healthy adult females exhibiting normal foraging migrations.

In addition to animal deployments, we evaluated the accuracy of GPS positions on land in a stationary test of four Fastloc GPS tags. Tags were placed within a one-meter square with an open view of the sky at Long Marine Lab in Santa Cruz, California, USA to collect positions for 24 h.

### Data Handling

Each deployment yielded an ARGOS track and an archived GPS track. All tracks were truncated to remove periods on land immediately after attaching the tags and immediately prior to removing the tags. ARGOS data were downloaded daily to a local database. ARGOS occasionally updated the location class of previously downloaded locations and these changes were automatically incorporated into our database. ARGOS tracks were filtered using a 100 km·hr^−1^ speed filter to remove extreme outliers as we were interested in the performance of the ARGOS system and not the performance of filtering algorithms. This outlier filter removes only the most obviously erroneous locations as a simple speed filter for pinnipeds typically assumes a maximum swim velocity of less than 10 km·hr^−1^. As the GPS tracks were our estimate of “true” position and since occasional errors are generated from the GPS tracks, we used a conservative filter for all GPS tracks: 10 km·hr^−1^ and 170-degree angle.

To determine the accuracy of ARGOS location estimates, we first identified all ARGOS locations obtained within five minutes of any GPS position. Next, the “true” position of the animal at the exact time of the ARGOS uplink was estimated via linear interpolation between the two neighboring GPS positions [38]. This procedure ensures every estimate of ARGOS error was based on a GPS position proximate in both space and time to the true position of the animal. We then calculated several error statistics from each ARGOS-GPS pair: great-circle distance, bearing, longitude component of error, and latitude component of error. The magnitudes of distance errors were fitted to a lognormal distribution for each ARGOS location class using a maximum likelihood approach.

Argos location quality proportions were compared between species using a G-test. Location errors were log transformed and modeled using a linear mixed effects model (SAS 9.1) with individual animal as a random effects subject term and species and location quality as fixed effects. Model residuals were assessed for approximate normality. The ARGOS-GPS data pairs used in this analysis are available as supplementary material to allow further analysis of these data (S1).

All analyses were performed using custom-written software in Matlab R2007a (The Mathworks Natick, MA) and Systat 12 (Chicago, IL).

## Results

**Figure 1:**
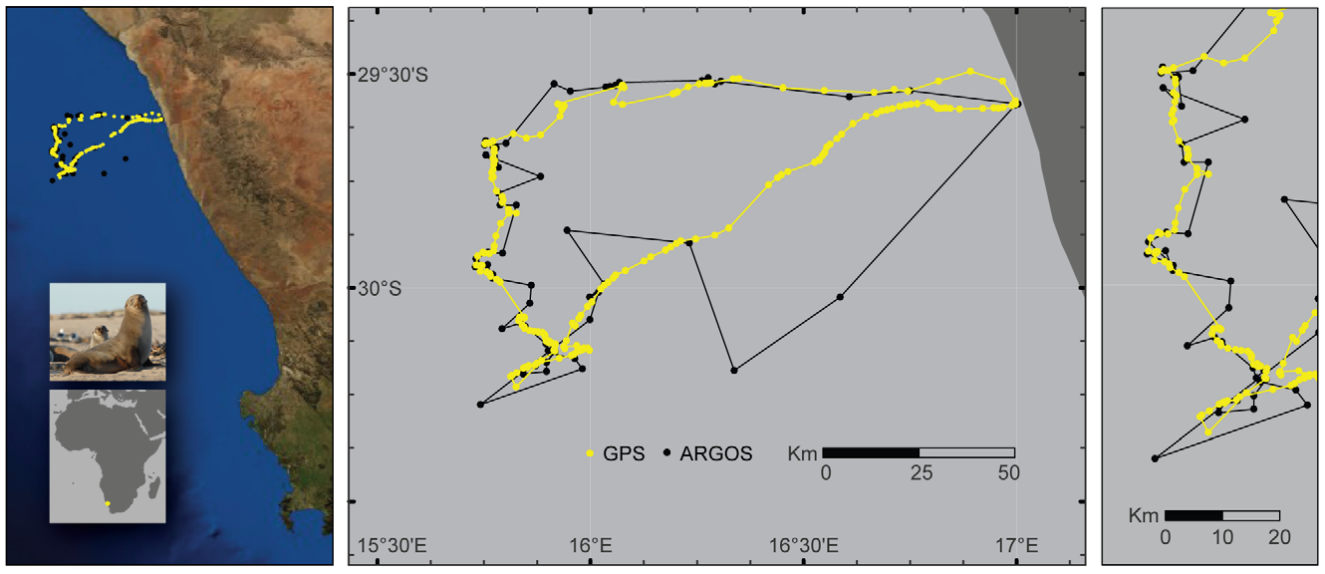
A representative track of a female Cape fur seal off the western coast of South Africa obtained using ARGOS (black) or Fastloc GPS (yellow) tags. All filtered locations are presented in this figure, while the comparison between ARGOS and GPS used only locations that were obtained within 5 min of each other.

Matched ARGOS/GPS tracks were obtained from 28 individuals comprising five species of pinnipeds. A total of 7,318 ARGOS positions and 27,046 GPS positions were collected. Of these, 1,105 ARGOS positions were obtained within five minutes of a GPS position and were retained for further analysis. The mean time between ARGOS-GPS pairs was on average 1.16 days (median 0.58 days) with less than 1% (81) of these pairs occurring within an hour of each other. An example of the data used to compare ARGOS against GPS locations is provided for a Cape fur seal (Fig. 1). Error estimates (great-circle distance, bearing, longitude component of error, and latitude component of error) were calculated for each ARGOS position and are available as supplementary material. Mean ARGOS error for all LC combined was 19.44 km with a standard deviation of 116.89 km (median error 1.91 km). The mean ARGOS error, standard deviation and sample size for each of the ARGOS location classes are summarized for each species in Table 1. Location quality proportions varied significantly between species (G^2^ = 525.3, df = 28, p<0.001). When controlled for location quality, location error varied between species (F_4,1071_ = 7.8, p<0.001). Post-hoc comparison of least square means revealed that irrespective of location quality, elephant seals had higher errors than all of the otariid species and Australian fur seals had higher errors than the other otariids. (Sidak adjustment, p<0.001).

**Table 1:** The mean ARGOS error in kilometers along with the standard deviation and sample size for each of the ARGOS location classes summarized for each of the 5 species of pinnipeds.

	Error in km						
	LC-3	LC-2	LC-1	LC-0	LC-A	LC-B	LC-Z
Cape fur seal	1.38	1.08	1.13	3.03	6.58	6.98	1.50
std dev	±1.62	±0.92	±0.99	±2.52	±8.77	±9.30	-
n	2	5	19	21	38	27	1
California sea lion	0.60	0.95	1.05	3.87	4.41	7.67	89.70
std dev	±0.56	±1.00	±1.01	±5.59	±6.47	±10.80	±28.47
n	14	71	184	102	81	85	3
Australian fur seal	0.34	0.75	2.18	10.41	19.76	97.71	9.71
std dev	±0.20	±0.512	±1.40	±13.12	±35.96	±115.81	-
n	5	9	11	13	5	10	1
Galapagos sea lion	0.26	0.94	1.04	4.34	5.22	20.50	31.87
std dev	±0.18	±1.81	±1.09	±3.350	±4.63	±30.85	±20.36
n	12	19	36	23	16	8	3
Northern eseal	-	0.91	3.04	3.99	101.17	49.23	164.45
std dev		±0.50	0.00	±5.83	±341.94	±189.33	±236.35
n	-	3	1	8	52	206	11

**Figure 2:**
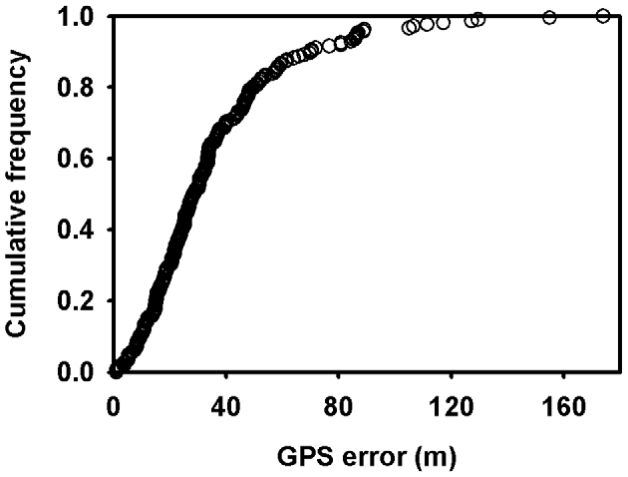
The cumulative frequency of GPS errors from a stationary test of fast-loc GPS tags (N = 257) are shown.

The GPS positions obtained from the 4 tags used in the stationary test had a mean error of 35.69 m with 95% of GPS positions falling within 86 m of the mean geographic location of all positions (Fig. 2).

**Figure 3:**
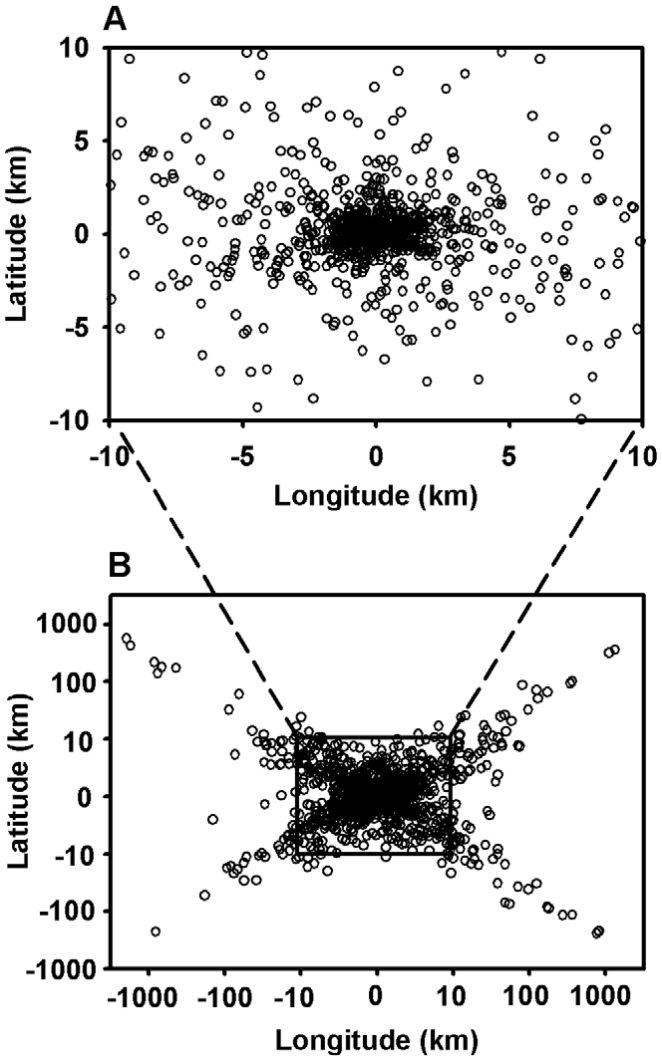
The ARGOS error measured as offset from the “true” position of the animal as compared to GPS locations are given in this figure. Note the logarithmic scaling of the lower panel and linear scaling of the upper panel.

**Figure 4:**
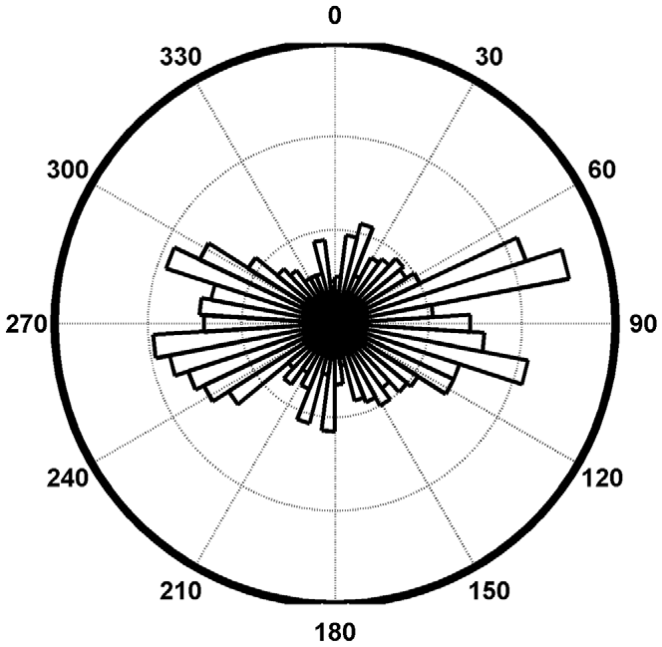
The frequency distribution of the error in ARGOS locations are provided in this figure. East/west offsets were frequent relative to north/south offsets.

ARGOS position errors from the animal borne tests were centered on “true” locations but extreme outliers were numerous and did not occur in a circular-uniform distribution (Fig. 3). Similarly, for the direction frequency of ARGOS errors, the east/west (longitudinal) error component was large relative to the north/south component (latitudinal) (Fig. 4).

**Figure 5:**
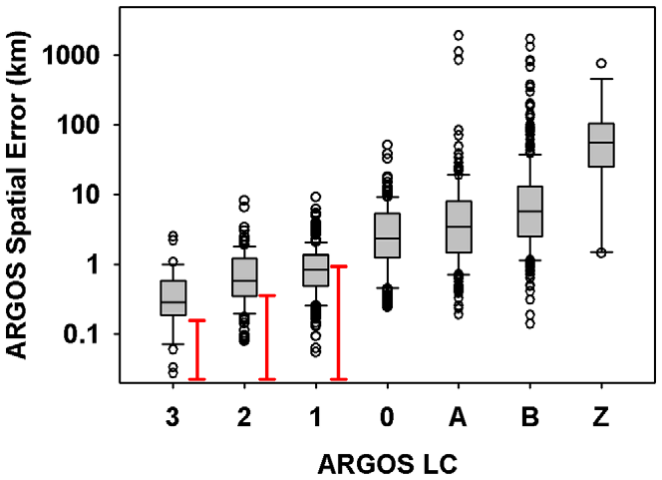
A box and whisker plot of the ARGOS error associated with each location class is given in this figure. Red bars represent the 68% error as estimated by ARGOS. Note the log-scaling of the y-axis.

**Figure 6:**
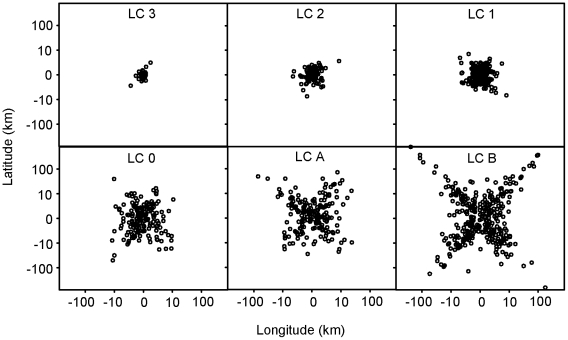
This figure provides the ARGOS error as standardized to a fixed location (N = 1105). Data are log+1 transformed (negative values were reflected before transformation).

We found larger than expected errors for the three LCs (3, 2, and 1) for which error estimates are provided by ARGOS (Fig. 5). The remaining LCs exhibit a stepwise increase in error and a substantial reduction in accuracy for LC-Z locations (Fig. 5). Two-dimensional visualization of the ARGOS errors show increased dispersion with lower quality locations but also a distinct directional offset in the extreme outliers (Fig. 6). This was particularly apparent in LC-A and LC-B locations. As the data were clearly skewed we chose to fit the distance errors to lognormal distributions for each LC (Table 2).

**Table 2:** Parameters describing lognormal probability distribution for the distance (km) of ARGOS error for each location class.

ARGOS LC	Sample Size	μ	σ
3	33	−1.198	1.02
2	107	1.026	0.693
1	251	1.199	0.712
0	167	1.381	0.521
A	192	1.622	0.506
B	336	1.623	0.612

**Figure 7:**
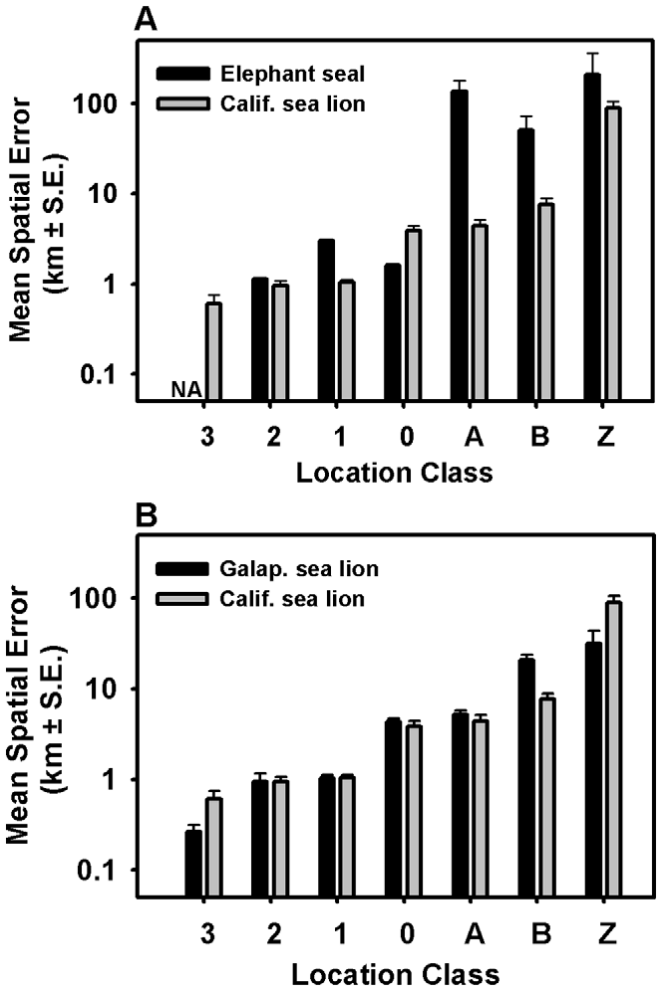
**A.** The mean spatial error for each location class is provided for two species that exhibit different surfacing behaviors. Spatial errors from northern elephant seals were large relative to California sea lions, particularly for “A” and “B” locations, which were the most common for the northern elephant seals. **B.** The mean spatial error for each location class is provided for two closely related species at different latitudes. These data show a negligible impact of latitude on ARGOS location error.

The species' diving behavior was also an important factor in the relative location error. Species with similar behaviors had similar location errors (Fig. 7 Table 3), whereas species with dissimilar at-sea behaviors had divergent location errors. For example, location error was smaller for species that spend more time on the surface and make shorter duration dives (sea lions and fur seals; 2.2–4.0 min) than species that make long dives followed by short surface intervals (elephant seals; 21.3 min) (Fig. 7). It is important to note that behavior likely impacts both the relative proportion of the LCs and the error within each LC.

**Table 3:** Summarizes the species, sample size, location and duration of deployment and the data obtained for that species, along with data on their diving behavior.

Species	Sample size	Mean latitude	Mean longitude	Deployment duration (weeks)	# ARGOS positions	# GPS positions	# positions used in analysis	Mean ARGOS LC	Mean error (km) light filter	Mean error (km) no filter	Mean dive duration (min)	reference
California sea lion	9	33.92	−119.970	8	4746	9219	540	1-0 (3.80)	3.59	9.49	2.2	Kuhn 2006
Galapagos sea lion	4	−0.764	−90.413	2	344	1705	117	1-0 (3.41)	4.29	88.56	4	Villegas et al. 2008
Cape fur seal	6	−29.802	16.267	4	917	1597	113	0-A (4.53)	4.72	18.67	2.5	Harrison et al (unpublished)
Australian fur seal	3	−39.357	146.304	2	165	919	54	1-0 (3.70)	23.21	92.74	2.9	Arnould and Hindell, 2000
Northern elephant seal	6	42.739	−135.902	12	1146	13606	281	A-B (5.74)	61.38	173.11	21.3	Leboeuf et al 2000
Stationary test of GPS This study	4	36.949	−122.066	–	–	257	–	–	–	–	–	–

## Discussion

We assessed the accuracy of ARGOS locations for free-ranging pinnipeds by comparing ARGOS locations to Fastloc GPS locations (matched closely in time, but also interpolated for improved accuracy), which we treated as true locations. Although our on-land stationary test of Fastloc GPS showed greater location error than that achieved with a commercial handheld GPS unit (e.g. 3m Garmin Inc., Olathe, KS), the accuracy was still 4 times greater than the best ARGOS position estimates (Fastloc GPS 36 m; ARGOS LC-3 = 150 m). We, therefore, conclude that Fastloc GPS provides an appropriate method to assess ARGOS error on free-ranging marine animals.

Incorporating ARGOS error in track analysis is certainly not a novel idea. Attempts to measure this error empirically have ranged in complexity from simple stationary land tests to acoustic tracking of free-ranging satellite-tagged seals and turtles (Table 4). However, these studies are problematic for four reasons: (1) The methodology used in most of these studies failed to capture several sources of ARGOS location error; (2) the studies that properly account for these additional sources of ARGOS error suffer from very small sample sizes and thus cannot adequately describe the error for some ARGOS LCs; (3) many of the studies report either mean errors or 68^th^ percentile errors in lieu of a full distribution (this is problematic for the implementation of results in modeling exercises and may result in the application of an inappropriate distribution); and (4) the extreme variability in estimates of ARGOS error within a particular LC between studies is problematic for proper implementation. For example, published values for LC-0 error span an entire order of magnitude (Table 4). The lack of congruence between previous studies may also be due to a combination of low sample sizes, variability in animal behavior, and study protocol. Nevertheless, it is re-assuring that the two studies that arguable employ the most realistic validation method (Fastloc GPS) obtained the most similar error estimates (Table 4; this study and [35] ). This is somewhat unexpected as we might have expected better accuracy from ARGOS transmitters in a tethered float [35] compared to tags mounted on the head or mid back (this study). Regardless, our study is one of the first to empirically determine ARGOS location error from marine animal-borne satellite tracking units where the tag was directly attached to the animal, thereby appropriately accounting for the combined effects of factors known to influence the accuracy of position estimates.

**Table 4:** Comparison of ARGOS error from different studies. (If two values are present in a cell, this indicates ‘latitude/longitude’).

Source	Method	Statistic	LC-3	LC-2	LC-1	LC-0	LC-A	LC-B
ARGOS	Theoretical	68th percentile	0.15/0.15	0.35/0.35	1.00/1.00	–	–	–
**This study**	**on animals, at sea**	**Mean**	**0.487**	**0.939**	**1.110**	**4.342**	**31.512**	**36.077**
**This study**	**on animals, at sea**	**68th percentile**	**0.486**	**1.011**	**1.201**	**4.182**	**6.185**	**10.276**
**This study**	**on animals, at sea**	**68th percentile Lat/long**	**0.225/0.340**	**0.468/0.729**	**0.574/0.879**	**1.795/2.855**	**2.788/4.373**	**4.642/8.253**
Hazel 2009	on animals, at sea	68^th^ percentile	0.482	0.785	1.430	5.179	8.072	11.484
Goulet et al. 1999	stationary test on land	68th percentile	–	–	1.335	43.799	–	–
Keating et al. 1991	stationary test on land	68th percentile	0.361	0.903	1.188	–	–	–
Mate et al. 1997	stationary test on land	68th percentile	0.3	0.9	2.3	7.5	–	–
McConnell et al. 1992b	on animals, on land	68th percentile	–	1.022	2.238	3.792	–	–
Britten et al. 1999	on animals, on land	68th percentile	–	–	–	11.5	6.8	98.5
Vincent et al. 2002	on animals, study pool	68th percentile Lat/long	0.157/0.295	0.259/0.485	0.494/1.021	2.271/3.308	0.762/1.244	4.596/7.214
Burns and Castellini 1998	on animals, on ice	Mean Lat/long	–	–	4.1/2.5	8.8/5.5	–	–
Boyd et al. 1998	Stationary test on land	Mean	1.228	1.115	1.566	3.779	18.843	22.841
Hays et al. 2001	stationary test on land	Mean Lat/long	0.12/0.32	0.28/0.62	1.03/1.62	4.29/15.02	1.39/0.81	5.23/7.79
Le Boeuf et al. 2000	on animals, on land	Mean	0.8	1.4	2.7	9.3	28.3	48.4
Nicholls et al. 2007	stationary test on land	Mean	0.5	0.7	1.7	16	5	38
White and Sjoberg 2002	on animals, at sea	Grand mean	–	–	4.434	5.349	6.477	45.345

Note: Percentile and mean values are presented.

There is no question that GPS tracks are superior to those obtained with ARGOS (Fig 1). We applied only a basic speed filter to create the tracks in Figure 1 and, with the more sophisticated filtering and smoothing algorithms now available, ARGOS tracks can be considerably improved [24], [28], [39]. However, our estimates of ARGOS error were significantly larger than those provided by ARGOS (approximately 300% greater for LC-3 and LC-2). Although published estimates of ARGOS error vary widely (Table 4), estimates from this study show larger than expected errors for the better location classes and smaller than expected errors for poor location classes.

Inspection of the ARGOS error distributions revealed two features of interest in the direction of the offset: 1) errors are heavily biased in the east/west axis (Fig. 3); and 2) the outliers do not occupy a circular uniform distribution around the true location but instead are preferentially found on the diagonals (Fig. 5 LC-A & LC-B). This pattern has been previously reported (22, 34) and is due to the polar orbit of the ARGOS satellite. The non-uniformity of ARGOS errors around the true location has direct impacts to existing modeling techniques [26], [28], [39]. Typically points are sampled in one of two ways: 1) an X-Y approach by sampling from a distribution of latitudinal error and separately from a distribution of longitudinal error; or 2) a polar approach by sampling from a distribution of straight-line distance and then from a uniform distribution for direction. Both methods do not adequately account for the observed distribution of outliers (exemplified in LC-A and LC-B). We recommend a polar approach where both distance and angle distributions are defined by empirical ARGOS error data to properly account for the unique two-dimensional shape of the error distributions. These can be created using the supplementary material provided (S1).

Species-specific differences in track quality are predominantly due to variation in surfacing intervals and dive durations shifting the frequency of LCs. Hays et al. (2001) suggest that ARGOS error, even within a particular LC, is highly variable and likely dependent upon animal diving behavior. Ideally, ARGOS error should be assessed independently for each tracking study. While this is rarely possible, we have provided data derived from different species with a range of surfacing behaviors (Table 1 & 3). Not surprisingly, location errors were similar for the two sea lion species that swim on the surface and have similar diving patterns (Table 1, Fig. 7B). In comparison, northern elephant seal ARGOS tracks were characterized by considerably greater location error (Table 1, Fig 7A). This result is not unexpected considering that the dive duration of elephant seals is about as long as the time an ARGOS satellite is overhead (20 min), meaning that all of the location information will most likely be transmitted during a single 2 to 3 minute surface interval. In contrast, fur seals and sea lions are likely to surface on multiple occasions while the ARGOS satellite is overhead, providing for more uplinks. In addition, an ARGOS tag attached to an elephant seal is likely to undergo a greater range of temperatures (4–15°C), and more frequent temperature changes, than a tag attached to either a fur seal or sea lion. This is because the deep diving nature of elephant seals will take the tag through a greater range of ambient temperatures and, once at the surface, the satellite tag is likely to warm rapidly after spending 21 min at depths between 400–600m. Other investigators may want to create their own error distributions using an appropriate subset of the ARGOS error data provided here as supplementary material (S1), considering species-specific diving behavior (Table 3).

Basic descriptions of movements over large spatial scales do not require a detailed understanding of ARGOS location error but the implementation of quantitative analytical tools that derive behavioral states requires accurate knowledge of the error structure [28], [39], [40]. This is particularly true for analyses at or near the spatio-temporal resolution of the tracking data itself, as these analyses will be particularly sensitive to estimates of error and may provide spurious interpretations if inaccurate estimates are used. Several studies highlight the utility of error distributions in both track “filtering” and behavioral interpretation [26], [40], [41], [42], [43]. New track improvement algorithms bypass traditional filtering techniques (i.e. simple removal of points) in favor of using all positions weighted by error estimates of the corresponding location class. Several studies use this approach in either a frequentist or Bayesian framework to re-create a more accurate track [28], [39]. In addition, these methods are used to extrapolate the likely behavioral state of the tagged animal [39], [44].

Although Fastloc GPS drastically improves the quality of tracking data of diving marine animals and will facilitate analyses at smaller spatio-temporal scales, the abundance of historic ARGOS tracking records and relative ease of ARGOS tracking (i.e. no recapture requirement) will ensure a continued demand for analytical approaches using these data. It is essential that these analyses utilize realistic error distributions to recreate a more accurate track and ensure estimates of behavioral states are not driven by noise in the position estimates. Future work should investigate the impact of track quality parameters, such as accuracy and frequency of positions, on biological interpretation of animal movement data.

## Supporting Information

Data S1 The supplemental data consists of an error estimate for each ARGOS location within five minutes of any GPS position. The supplemental file is broken into separate files for each of the five species of pinnipeds. For each species file the individual animals are identified by “Animal ID” and the great-circle distance, bearing, longitude component of error, and latitude component of error that was calculated for each ARGOS location are provided along with the ARGOS location class (LC) given for that location and the associated GPS residual value. A digital copy of these data are available from the corresponding author.
(0.14 MB PDF)
