# Supplementary material for: Accuracy of ARGOS Locations of Pinnipeds at-Sea Estimated Using Fastloc GPS
Source: PLoS One. 2010 Jan 15;5(1):e8677. doi: 10.1371/journal.pone.0008677 (PMC2806907; doi:10.1371/journal.pone.0008677)
Supplement: Data S1 — The supplemental data consists of an error estimate for each ARGOS location within five minutes of any GPS position. The supplemental file is broken into separate files for each of the five species of pinnipeds. For each species file the individual animals are identified by “Animal ID” and the great-circle distance, bearing, longitude component of error, and latitude component of error that was calculated for each ARGOS location are provided along with the ARGOS location class (LC) given for that location and the associated GPS residual value. A digital copy of these data are available from the corresponding author. (0.14 MB PDF) [file pone.0008677.s001.pdf]

Species	Animal ID	error (km)	angle	lat error(km)	lon error (km)	GPS residual	ARGOS LC
Galapagos sea lion	19	1.5373	138.1207	1.1446	-1.0263	15.6	0
Galapagos sea lion	19	5.4969	278.3526	-0.7985	5.4386	14.3	0
Galapagos sea lion	19	7.5565	60.2603	-3.7485	-6.5612	2	0
Galapagos sea lion	19	0.2392	166.7388	0.2328	-0.0549	12.5	3
Galapagos sea lion	19	36.6264	102.439	7.8882	-35.7667	1.1	B
Galapagos sea lion	19	1.2978	139.6045	0.9884	-0.841	13.3	1
Galapagos sea lion	19	0.3934	86.9352	-0.021	-0.3929	10.7	1
Galapagos sea lion	19	0.8524	304.9264	-0.488	0.6989	9.1	1
Galapagos sea lion	19	4.4369	278.9589	-0.691	4.3828	0.8	A
Galapagos sea lion	19	0.9654	277.0967	-0.1193	0.958	12.5	2
Galapagos sea lion	19	12.9292	32.9504	-10.8495	-7.0323	14.2	0
Galapagos sea lion	19	11.0073	139.2815	8.3426	-7.1805	1.7	0
Galapagos sea lion	19	1.1235	232.0088	0.6916	0.8854	7.7	2
Galapagos sea lion	19	15.299	59.4693	-7.7721	-13.1779	1.1	A
Galapagos sea lion	19	3.8785	212.6013	3.2674	2.0897	0.2	B
Galapagos sea lion	19	0.4658	169.0732	0.4574	-0.0883	0.3	0
Galapagos sea lion	19	0.3243	92.7073	0.0153	-0.324	27.8	1
Galapagos sea lion	19	1.1609	156.8905	1.0678	-0.4557	11	1
Galapagos sea lion	19	3.9713	268.8302	0.0811	3.9705	3	A
Galapagos sea lion	19	2.068	171.026	2.0427	-0.3226	14.3	0
Galapagos sea lion	19	0.3246	65.0464	-0.137	-0.2943	0.2	A
Galapagos sea lion	19	11.3076	192.6217	11.0343	2.4709	1.1	A
Galapagos sea lion	19	3.4516	132.4066	2.3277	-2.5486	12.4	1
Galapagos sea lion	19	9.2919	307.6987	-5.6821	7.3521	12.4	B
Galapagos sea lion	20	1.5554	16.119	-1.4942	-0.4318	12.7	0
Galapagos sea lion	20	3.9187	5.4427	-3.9011	-0.3717	4	0
Galapagos sea lion	20	0.5964	337.4112	-0.5506	0.2291	8.6	1
Galapagos sea lion	20	1.8454	327.9424	-1.564	0.9795	12.7	0
Galapagos sea lion	20	0.4564	93.7328	0.0297	-0.4554	3.8	3
Galapagos sea lion	20	0.3507	131.4262	0.232	-0.2629	9.6	2
Galapagos sea lion	20	0.4332	263.5381	0.0487	0.4304	15.8	0
Galapagos sea lion	20	1.0264	129.5388	0.6534	-0.7915	11.2	2
Galapagos sea lion	20	8.2972	288.729	-2.6642	7.8578	3.2	0
Galapagos sea lion	20	3.5998	250.8304	1.182	3.4002	23.2	1
Galapagos sea lion	20	5.4498	314.9505	-3.8502	3.8569	9.1	1
Galapagos sea lion	20	8.371	144.1996	6.7894	-4.8967	17.9	Z
Galapagos sea lion	20	5.4027	144.4811	4.3973	-3.1388	5.5	B
Galapagos sea lion	20	1.8155	259.9427	0.317	1.7876	18	1
Galapagos sea lion	20	4.3042	86.0041	-0.3	-4.2937	1.4	0
Galapagos sea lion	20	1.1969	106.3165	0.3363	-1.1487	2.6	1
Galapagos sea lion	20	4.0097	155.218	3.6405	-1.6807	0.2	B
Galapagos sea lion	20	1.2016	95.8915	0.1233	-1.1952	0.3	1
Galapagos sea lion	20	7.3306	148.6128	6.2579	-3.8179	28	0
Galapagos sea lion	20	0.93	62.9297	-0.4232	-0.8282	0.3	1
Galapagos sea lion	20	0.8446	107.3295	0.2516	-0.8062	0.7	1
Galapagos sea lion	20	0.0959	213.4318	0.08	0.0528	0.3	2
Galapagos sea lion	20	1.2568	20.9854	-1.1735	-0.4501	3.8	1
Galapagos sea lion	20	1.5053	151.9505	1.3285	-0.7078	0.7	2
Galapagos sea lion	20	3.707	52.0548	-2.2795	-2.9234	0.8	A
Galapagos sea lion	20	3.8117	0.3317	-3.8117	-0.0221	0.1	0
Galapagos sea lion	20	2.8206	129.5418	1.7957	-2.1752	0.2	0
Galapagos sea lion	20	6.6079	188.1308	6.5414	0.9346	6.9	0
Galapagos sea lion	20	2.2178	49.7444	-1.4331	-1.6926	1.5	A
Galapagos sea lion	21	4.3899	136.5898	3.189	-3.0168	12.7	0
Galapagos sea lion	21	1.8302	210.2895	1.5803	0.9231	27.1	B
Galapagos sea lion	21	0.2446	261.9908	0.0341	0.2422	2.4	1
Galapagos sea lion	21	1.2682	21.1073	-1.1832	-0.4567	10.4	A
Galapagos sea lion	21	0.2222	112.2364	0.0841	-0.2057	20	3
Galapagos sea lion	21	0.1293	42.7751	-0.0949	-0.0878	15.1	3
Galapagos sea lion	21	1.0283	260.5371	0.1691	1.0144	10.6	2
Galapagos sea lion	21	0.5411	70.3421	-0.182	-0.5096	15.5	1
Galapagos sea lion	21	0.134	338.6583	-0.1249	0.0488	4.9	1
Galapagos sea lion	21	0.2255	55.5576	-0.1276	-0.186	5.3	2
Galapagos sea lion	21	42.9403	260.0496	7.4178	42.2943	3.2	Z

Galapagos sea lion	21	91.6702	109.8368	31.1002	-86.2304	0.1	B
Galapagos sea lion	21	44.3046	106.8509	12.8411	-42.4022	1	Z
Galapagos sea lion	21	1.5053	71.2646	-0.4835	-1.4256	0.6	1
Galapagos sea lion	21	11.2697	122.0361	5.9779	-9.5535	9.7	B
Galapagos sea lion	21	0.4834	83.3779	-0.0557	-0.4802	22.9	2
Galapagos sea lion	21	0.2515	134.9695	0.1778	-0.178	9.2	2
Galapagos sea lion	21	0.418	219.2067	0.3239	0.2643	12.6	1
Galapagos sea lion	21	0.1251	220.6351	0.0949	0.0815	0.5	3
Galapagos sea lion	21	0.4617	18.8223	-0.437	-0.1489	0.9	1
Galapagos sea lion	21	0.3443	348.1289	-0.3369	0.0708	1.1	2
Galapagos sea lion	21	0.0918	24.0446	-0.0838	-0.0374	1.8	1
Galapagos sea lion	21	8.2246	198.675	7.7915	2.6335	0.7	2
Galapagos sea lion	21	0.0332	57.3062	-0.0179	-0.028	0.5	3
Galapagos sea lion	21	0.5325	347.7443	-0.5204	0.113	0.7	1
Galapagos sea lion	21	0.9859	91.0474	0.018	-0.9858	0.2	0
Galapagos sea lion	21	0.1458	291.5997	-0.0537	0.1356	2	1
Galapagos sea lion	21	0.5961	359.1391	-0.596	0.009	6.2	1
Galapagos sea lion	21	4.6828	55.2325	-2.6704	-3.8468	6	0
Galapagos sea lion	21	0.2853	236.7277	0.1565	0.2385	3.1	3
Galapagos sea lion	21	3.0191	180.419	3.019	0.0221	5	A
Galapagos sea lion	22	0.4459	11.2455	-0.4374	-0.087	11.6	3
Galapagos sea lion	22	0.2761	126.9129	0.1659	-0.2208	4	3
Galapagos sea lion	22	0.1651	310.4479	-0.1071	0.1256	25.3	3
Galapagos sea lion	22	3.6968	266.6374	0.2168	3.6905	5.7	A
Galapagos sea lion	22	1.5141	103.8768	0.3631	-1.4699	10.8	1
Galapagos sea lion	22	0.1442	318.0762	-0.1073	0.0963	7.5	1
Galapagos sea lion	22	0.0889	272.7662	-0.0043	0.0888	9	3
Galapagos sea lion	22	13.1762	135.4153	9.3842	-9.2492	1	A
Galapagos sea lion	22	0.8083	286.8964	-0.2349	0.7734	15.7	1
Galapagos sea lion	22	0.4636	304.5947	-0.2632	0.3817	2.4	1
Galapagos sea lion	22	0.2464	193.0404	0.2401	0.0556	0.6	2
Galapagos sea lion	22	1.086	195.1824	1.0481	0.2844	0.5	1
Galapagos sea lion	22	0.6652	136.0193	0.4786	-0.4619	11.6	A
Galapagos sea lion	22	1.02	99.0644	0.1607	-1.0073	11.7	1
Galapagos sea lion	22	0.3152	127.193	0.1906	-0.2511	3.8	1
Galapagos sea lion	22	0.8593	201.5339	0.7993	0.3154	0.4	1
Galapagos sea lion	22	1.0549	251.51	0.3345	1.0004	0.3	1
Galapagos sea lion	22	0.0796	78.1794	-0.0163	-0.0779	10.7	2
Galapagos sea lion	22	8.4261	109.2545	2.7786	-7.9548	5.8	A
Galapagos sea lion	22	0.1895	304.1869	-0.1065	0.1567	13	A
Galapagos sea lion	22	7.0076	304.5001	-3.9692	5.7751	0.4	A
Galapagos sea lion	22	0.1722	179.4362	0.1722	-0.0017	8.4	2
Galapagos sea lion	22	4.7704	12.694	-4.6538	-1.0483	10.7	0
Galapagos sea lion	22	0.6029	33.2757	-0.5041	-0.3308	2.7	2
Galapagos sea lion	22	0.232	118.0933	0.1093	-0.2047	0.8	2
Galapagos sea lion	22	0.4168	227.8633	0.2796	0.3091	1	2
Galapagos sea lion	22	1.4057	352.2686	-1.3929	0.1891	5.6	0
Galapagos sea lion	22	0.2153	292.9451	-0.0839	0.1983	4.3	1
Galapagos sea lion	22	0.835	298.9408	-0.4041	0.7308	0.5	1
Galapagos sea lion	22	0.6775	49.7659	-0.4376	-0.5172	3.4	3
Galapagos sea lion	22	4.8242	114.9841	2.0375	-4.3727	3.6	A
Galapagos sea lion	22	1.6757	277.347	-0.2143	1.6619	0.8	0
Galapagos sea lion	22	0.5174	107.812	0.1583	-0.4926	1.2	2

Species	Animal ID	error (km)	angle	at error(km	n error (km	PS residu	ARGOS LC
California sea lion	7	2.2423	295.3506	-0.9598	2.0264	12.1	1
California sea lion	7	3.066	56.6638	-1.6846	-2.5615	19.7	0
California sea lion	7	0.7138	240.9711	0.3464	0.6241	8.5	1
California sea lion	7	0.419	111.1953	0.1515	-0.3906	1.4	2
California sea lion	7	0.8812	223.0179	0.6443	0.6012	9.9	1
California sea lion	7	0.513	145.867	0.4247	-0.2879	2.1	1
California sea lion	7	1.985	292.8873	-0.7718	1.8288	13.3	1
California sea lion	7	0.9073	75.9993	-0.2195	-0.8804	7.8	2
California sea lion	7	2.0571	280.488	-0.3742	2.0227	13.8	1
California sea lion	7	0.9839	232.5387	0.5985	0.781	24.4	2
California sea lion	7	1.1768	93.7914	0.0779	-1.1742	8.1	0
California sea lion	7	0.3327	44.5481	-0.2371	-0.2334	1	2
California sea lion	7	0.7151	358.3018	-0.7148	0.0212	8.2	B
California sea lion	7	0.1673	117.6282	0.0776	-0.1482	12.3	1
California sea lion	7	0.9481	247.5129	0.3627	0.876	17.8	1
California sea lion	7	2.4237	214.9086	1.9877	1.387	0.8	B
California sea lion	7	0.5921	124.3365	0.334	-0.4889	9.8	1
California sea lion	7	0.3063	108.8633	0.099	-0.2898	10	1
California sea lion	7	0.0624	178.8755	0.0624	-0.0012	1.4	1
California sea lion	7	28.4839	89.1378	-0.385	-28.4807	29.5	A
California sea lion	7	0.2574	100.55	0.0471	-0.253	4.5	1
California sea lion	7	0.9529	274.5128	-0.0749	0.95	20	1
California sea lion	7	2.0657	268.5772	0.0515	2.0651	9.5	1
California sea lion	7	1.1558	161.2515	1.0945	-0.3715	6.4	B
California sea lion	7	2.2609	67.7406	-0.8562	-2.0924	1.8	B
California sea lion	7	4.978	17.9791	-4.7348	-1.5366	12.7	B
California sea lion	7	3.4563	293.818	-1.3953	3.162	11.8	0
California sea lion	7	2.2036	273.8207	-0.1466	2.1987	14.3	A
California sea lion	7	0.6479	322.3285	-0.5128	0.3959	28.1	0
California sea lion	7	11.234	65.2257	-4.702	-10.2001	7	A
California sea lion	7	1.2373	266.2388	0.0812	1.2346	14.8	0
California sea lion	7	0.1577	230.9226	0.0994	0.1224	1.8	2
California sea lion	7	2.4915	268.9877	0.0443	2.4911	12.8	0
California sea lion	7	1.1868	316.7438	-0.8643	0.8133	14.8	A
California sea lion	7	1.8096	256.0108	0.4376	1.7559	15.2	1
California sea lion	7	0.2152	144.1725	0.1745	-0.126	3	2
California sea lion	7	0.6291	227.722	0.4232	0.4655	0.6	2
California sea lion	7	1.2317	153.1302	1.0988	-0.5567	18.3	2
California sea lion	7	5.0568	119.8006	2.5141	-4.3881	1.6	1
California sea lion	7	4.334	255.2997	1.1008	4.1921	9.1	A
California sea lion	7	2.0626	42.3225	-1.5249	-1.3888	12	1
California sea lion	7	0.1973	101.9	0.0407	-0.193	20.8	1
California sea lion	7	0.3738	205.4385	0.3376	0.1606	8.1	1
California sea lion	7	1.607	76.9008	-0.3641	-1.5652	8.6	0
California sea lion	7	1.2325	91.4496	0.0313	-1.2321	11.4	0
California sea lion	7	0.2192	220.9509	0.1656	0.1437	5.8	2
California sea lion	7	0.4829	237.2666	0.2611	0.4062	0.5	0
California sea lion	7	0.6331	158.379	0.5885	-0.2333	0.2	B
California sea lion	7	6.6219	65.34	-2.7609	-6.0179	14.7	B
California sea lion	7	0.4043	82.4864	-0.0529	-0.4008	5.4	3
California sea lion	7	2.8325	246.6892	1.1212	2.6013	5.5	1

California sea lion	7	0.616	218.1068	0.4847	0.3802	23.2	1
California sea lion	7	8.1405	346.4034	-7.9122	1.9137	10.2	0
California sea lion	7	1.7154	45.8542	-1.1947	-1.231	28.9	A
California sea lion	7	0.3094	233.1756	0.1855	0.2477	19	B
California sea lion	7	0.9522	106.7863	0.275	-0.9116	21.8	1
California sea lion	7	0.87	80.3135	-0.1463	-0.8576	6.4	3
California sea lion	7	1.7873	245.4031	0.7441	1.6251	11.6	1
California sea lion	7	3.1916	204.9345	2.8942	1.3455	11.5	B
California sea lion	7	0.2459	95.1955	0.0223	-0.2449	32.8	1
California sea lion	7	60.6347	251.293	19.625	57.4316	0.2	Z
California sea lion	7	0.9984	275.8138	-0.1011	0.9933	24.5	1
California sea lion	7	2.2001	351.1653	-2.174	0.3379	0.2	0
California sea lion	7	1.3402	300.4671	-0.6794	1.1551	3.3	1
California sea lion	7	1.8359	77.8682	-0.3857	-1.7949	0.5	1
California sea lion	7	0.0911	9.6299	-0.0899	-0.0152	2.9	2
California sea lion	7	1.265	209.185	1.1044	0.6168	4.4	0
California sea lion	7	0.3663	148.1633	0.3112	-0.1932	2.4	0
California sea lion	7	0.4867	34.8294	-0.3995	-0.2779	0.6	1
California sea lion	7	15.5391	226.0777	10.786	11.1925	8.2	B
California sea lion	7	0.381	161.1398	0.3606	-0.1232	4.5	2
California sea lion	7	0.9969	48.557	-0.6598	-0.7473	6.8	0
California sea lion	7	2.2149	196.7409	2.121	0.638	3.8	A
California sea lion	7	0.2447	259.8206	0.0433	0.2409	0.1	1
California sea lion	7	1.0117	11.6339	-0.9909	-0.204	18	2
California sea lion	7	2.1839	66.2678	-0.8787	-1.9992	16.7	B
California sea lion	7	61.1	62.1004	-28.4343	-53.9983	9	B
California sea lion	7	0.3736	308.0453	-0.2302	0.2942	27.1	2
California sea lion	7	5.2535	350.1549	-5.1761	0.8983	4.3	B
California sea lion	7	1.8566	127.8137	1.1384	-1.4668	19.6	A
California sea lion	7	1.4785	223.6743	1.0694	1.021	4.7	2
California sea lion	7	2.946	185.7453	2.9312	0.2949	3.9	B
California sea lion	7	2.0065	198.4292	1.9036	0.6343	17.7	0
California sea lion	7	0.6483	232.1882	0.3975	0.5122	16	1
California sea lion	8	0.0273	164.7459	0.0264	-0.0072	2.3	3
California sea lion	8	1.9069	241.3601	0.9141	1.6736	4.5	1
California sea lion	8	0.7116	175.0872	0.709	-0.0609	14.3	A
California sea lion	8	1.1137	64.0986	-0.4864	-1.0018	1.9	0
California sea lion	8	5.9367	305.2849	-3.4281	4.8461	1.1	B
California sea lion	8	14.2383	320.9295	-11.05	8.974	8.2	A
California sea lion	8	1.3709	99.6806	0.2306	-1.3514	17.7	1
California sea lion	8	6.1645	258.4263	1.2387	6.0392	13.8	B
California sea lion	8	1.5385	47.6318	-1.0367	-1.1367	5.4	1
California sea lion	8	12.5709	322.1786	-9.927	7.7085	21.9	A
California sea lion	8	1.2996	183.4025	1.2974	0.0771	12.8	0
California sea lion	8	1.31	199.0727	1.2381	0.4281	3.6	2
California sea lion	8	5.8519	346.5367	-5.6909	1.3624	9.2	0
California sea lion	8	0.6692	184.3906	0.6672	0.0512	10.1	2
California sea lion	8	15.6762	252.9528	4.6074	14.9875	10.5	B
California sea lion	8	0.6405	265.1871	0.0538	0.6382	10.8	1
California sea lion	8	0.4092	267.7777	0.0159	0.4089	6.8	B
California sea lion	8	0.5143	158.7004	0.4791	-0.1868	5.2	1
California sea lion	8	0.2745	250.1734	0.0931	0.2582	7.1	1

California sea lion	8	0.8251	262.4683	0.1082	0.818	8	0
California sea lion	8	2.3069	153.9573	2.0727	-1.0128	12.3	0
California sea lion	8	1.3626	131.1714	0.897	-1.0257	12.1	1
California sea lion	8	0.3583	276.3847	-0.0398	0.3561	9.1	0
California sea lion	8	0.8888	108.495	0.282	-0.8429	19.5	1
California sea lion	8	1.0889	255.5595	0.2716	1.0545	13.1	3
California sea lion	8	0.3823	58.4739	-0.1999	-0.3259	19.9	2
California sea lion	8	2.8546	137.0868	2.0909	-1.9437	5.5	B
California sea lion	8	1.8422	120.0607	0.9229	-1.5944	9.2	1
California sea lion	8	18.6556	146.1267	15.4948	-10.3978	7.4	B
California sea lion	8	0.5226	270.8791	-0.008	0.5225	5.4	1
California sea lion	8	6.6907	290.0371	-2.2904	6.2857	11.1	B
California sea lion	8	1.3994	275.1077	-0.1245	1.3939	9.5	0
California sea lion	8	1.1668	283.7006	-0.2763	1.1336	22.9	0
California sea lion	8	9.2294	259.0727	1.7538	9.062	10.3	0
California sea lion	8	0.4859	356.4718	-0.4849	0.0299	11.9	1
California sea lion	8	0.3234	299.2469	-0.158	0.2821	0.7	1
California sea lion	8	0.4883	146.9059	0.4091	-0.2666	14.5	1
California sea lion	8	0.4572	150.5841	0.3983	-0.2246	14.8	2
California sea lion	8	11.5517	265.126	0.9884	11.5099	4.7	B
California sea lion	8	0.8782	5.5243	-0.8741	-0.0845	3.5	A
California sea lion	8	2.1517	182.0615	2.1503	0.0774	17.7	1
California sea lion	8	2.7997	148.612	2.3901	-1.4581	11.4	A
California sea lion	8	6.7248	106.6978	1.9344	-6.4412	16.1	0
California sea lion	8	38.3201	281.0268	-7.2551	37.6127	22.9	A
California sea lion	8	0.5813	251.1217	0.1881	0.5501	0.8	2
California sea lion	8	2.4137	68.7959	-0.8728	-2.2503	8.8	0
California sea lion	8	1.8866	188.7652	1.8645	0.2875	12.2	1
California sea lion	8	0.6053	127.7479	0.3706	-0.4786	5.6	2
California sea lion	8	0.4267	278.9739	-0.0666	0.4215	24.5	1
California sea lion	8	0.5978	31.5084	-0.5097	-0.3124	5.1	1
California sea lion	8	0.9462	296.5499	-0.4229	0.8464	18.2	A
California sea lion	8	0.2498	131.4804	0.1655	-0.1872	9.5	1
California sea lion	8	1.5307	298.3286	-0.7263	1.3474	15	0
California sea lion	8	0.1746	218.7972	0.1361	0.1094	15.2	2
California sea lion	8	2.1191	20.7404	-1.9818	-0.7505	0.2	1
California sea lion	8	2.3638	91.1071	0.046	-2.3633	2.9	0
California sea lion	8	7.364	284.9568	-1.8979	7.1145	11.3	A
California sea lion	8	0.3475	98.4887	0.0513	-0.3437	9.9	1
California sea lion	8	1.5227	252.6739	0.4536	1.4536	17.8	1
California sea lion	8	0.6337	299.8062	-0.315	0.5499	2	1
California sea lion	8	1.7721	238.7606	0.9192	1.5152	4.3	2
California sea lion	8	1.5023	35.7436	-1.2193	-0.8776	8.8	1
California sea lion	8	1.0345	315.8634	-0.7424	0.7204	4.3	1
California sea lion	8	1.8807	344.9433	-1.8161	0.4886	2.1	B
California sea lion	9	3.0106	264.8577	0.2703	2.9985	5.1	0
California sea lion	9	1.2355	74.0917	-0.3386	-1.1882	14.2	1
California sea lion	9	12.5663	264.438	1.2261	12.5072	0.2	0
California sea lion	9	15.0341	263.4394	1.7294	14.9356	0.2	0
California sea lion	9	0.0545	20.7926	-0.0509	-0.0193	6.3	1
California sea lion	9	1.4779	81.2687	-0.2242	-1.4608	4.2	1
California sea lion	9	0.4879	120.9925	0.2513	-0.4183	4.7	1

California sea lion	9	1.7375	192.0813	1.6991	0.3637	1.4	A
California sea lion	9	0.2641	85.8896	-0.0189	-0.2634	0.5	1
California sea lion	9	2.2221	202.1227	2.0585	0.8368	4.1	2
California sea lion	9	3.5366	49.3872	-2.3017	-2.6847	0.8	0
California sea lion	9	2.6902	269.9309	0.0036	2.6902	0.5	B
California sea lion	9	0.6923	12.1122	-0.6769	-0.1453	14	1
California sea lion	9	0.5682	324.1594	-0.4606	0.3327	8.9	1
California sea lion	9	42.9971	261.3779	6.5406	42.5113	1.1	B
California sea lion	9	14.9186	22.1784	-13.8132	-5.6317	6.2	B
California sea lion	9	1.3716	235.5462	0.776	1.131	6.7	0
California sea lion	9	4.1402	349.1015	-4.0655	0.7828	4.1	B
California sea lion	9	1.6784	295.0938	-0.7117	1.52	0.4	1
California sea lion	9	1.4785	249.6964	0.5131	1.3867	3.9	1
California sea lion	9	0.3225	102.4297	0.0694	-0.3149	5.2	0
California sea lion	9	1.3641	244.6841	0.5834	1.2331	1.8	1
California sea lion	9	8.7691	185.4664	8.7293	0.8354	17.8	B
California sea lion	9	0.4248	83.329	-0.0493	-0.4219	2.2	1
California sea lion	9	21.3841	301.9007	-11.2832	18.1544	7.2	B
California sea lion	9	1.4132	341.721	-1.3419	0.4432	0.2	0
California sea lion	9	1.4864	12.2417	-1.4526	-0.3152	0.6	0
California sea lion	9	1.7763	284.2849	-0.4381	1.7214	0.1	B
California sea lion	9	1.5111	194.6443	1.462	0.382	18.1	1
California sea lion	9	1.7263	290.6864	-0.6097	1.615	0.1	0
California sea lion	9	0.1423	347.3379	-0.1388	0.0312	0.1	2
California sea lion	9	1.4762	121.2473	0.7658	-1.262	19.6	1
California sea lion	9	10.6075	113.5645	4.2456	-9.7229	10.1	B
California sea lion	9	3.9174	67.9953	-1.4671	-3.632	4.4	0
California sea lion	9	1.4201	28.8171	-1.2442	-0.6845	17.9	0
California sea lion	9	1.5764	59.7106	-0.795	-1.3612	17.9	0
California sea lion	9	1.3085	235.6849	0.7377	1.0808	1	1
California sea lion	9	1.6045	95.6268	0.1575	-1.5968	0.8	2
California sea lion	9	5.5347	8.9619	-5.4671	-0.8622	38	B
California sea lion	9	6.4113	271.8371	-0.2034	6.408	0.3	0
California sea lion	9	0.617	15.8622	-0.5935	-0.1686	24.5	1
California sea lion	9	10.8328	205.8049	9.7538	4.7156	6.4	0
California sea lion	9	9.1334	14.8979	-8.8261	-2.3482	5.5	0
California sea lion	9	0.4427	131.4006	0.2928	-0.3321	15.5	1
California sea lion	9	0.8581	36.8136	-0.687	-0.5142	0.5	1
California sea lion	9	1.4793	214.9017	1.2133	0.8464	0.2	2
California sea lion	9	3.0896	356.1113	-3.0825	0.2095	60.8	1
California sea lion	9	6.2088	283.7019	-1.4688	6.0322	2.6	1
California sea lion	9	4.9928	215.7395	4.053	2.9163	12.6	A
California sea lion	9	0.5036	198.4959	0.4776	0.1598	0.8	2
California sea lion	9	1.2875	161.4722	1.2208	-0.4091	7.8	0
California sea lion	9	1.2553	47.6644	-0.8454	-0.9279	0.8	0
California sea lion	9	5.4304	77.8092	-1.1453	-5.308	23.1	0
California sea lion	9	2.0393	335.0743	-1.8493	0.8595	9	A
California sea lion	9	1.3411	69.6248	-0.4668	-1.2571	7.1	2
California sea lion	9	1.2409	303.8193	-0.6906	1.0309	1.3	2
California sea lion	9	1.094	292.4631	-0.418	1.011	17	1
California sea lion	9	1.8168	51.0402	-1.1422	-1.4127	11.1	0
California sea lion	9	0.3348	208.5658	0.294	0.1601	15.4	1

California sea lion	9	1.2858	255.6395	0.319	1.2456	0.4	2
California sea lion	9	4.5456	352.5562	-4.5072	0.5889	0.4	0
California sea lion	9	0.7288	15.4683	-0.7024	-0.1944	1.9	1
California sea lion	10	0.2448	154.4174	0.2208	-0.1057	8.6	0
California sea lion	10	0.5054	270.2067	-0.0018	0.5054	18.7	1
California sea lion	10	0.7608	241.7136	0.3606	0.67	20.5	2
California sea lion	10	0.7787	128.0325	0.4798	-0.6134	7.7	1
California sea lion	10	0.6902	81.1952	-0.1056	-0.682	35.8	1
California sea lion	10	1.7911	297.5156	-0.8273	1.5885	8.8	A
California sea lion	10	1.7834	117.4708	0.8228	-1.5823	5.5	A
California sea lion	10	1.107	240.2533	0.5493	0.9611	1.7	A
California sea lion	10	0.7099	121.5058	0.371	-0.6053	10.9	1
California sea lion	10	0.8105	21.1261	-0.756	-0.2921	10.9	A
California sea lion	10	1.0984	270.7075	-0.0135	1.0983	5.5	B
California sea lion	10	4.169	278.2608	-0.5981	4.1258	10	0
California sea lion	10	0.3389	16.0629	-0.3256	-0.0938	1	1
California sea lion	10	0.3033	33.9157	-0.2517	-0.1693	17.8	1
California sea lion	10	0.3029	214.5645	0.2494	0.1718	13.1	2
California sea lion	10	3.5243	72.5031	-1.059	-3.3613	6.4	A
California sea lion	10	0.0936	160.839	0.0884	-0.0307	9.9	1
California sea lion	10	0.4878	204.3619	0.4444	0.2012	23.1	A
California sea lion	10	0.9044	257.8609	0.1902	0.8842	17.6	1
California sea lion	10	3.1197	153.1314	2.783	-1.4099	8.4	0
California sea lion	10	0.8353	55.0113	-0.4789	-0.6843	5.5	1
California sea lion	10	0.2541	102.3075	0.0542	-0.2483	9.2	0
California sea lion	10	2.5295	87.3748	-0.1155	-2.5268	0.3	B
California sea lion	10	1.4233	267.1226	0.0716	1.4215	18	0
California sea lion	10	1.7342	269.7535	0.0076	1.7342	14.1	1
California sea lion	10	0.4777	25.1868	-0.4323	-0.2033	8	A
California sea lion	10	0.1681	45.3125	-0.1182	-0.1195	0.4	2
California sea lion	10	2.1794	34.0682	-1.8053	-1.2209	13.4	0
California sea lion	10	0.4388	153.193	0.3917	-0.1979	0.7	0
California sea lion	10	0.2288	194.3324	0.2217	0.0566	16.4	1
California sea lion	10	0.3747	77.044	-0.084	-0.3651	16.4	1
California sea lion	10	0.4488	36.2275	-0.3621	-0.2653	2.7	2
California sea lion	10	1.4033	272.7103	-0.0663	1.4017	15.3	A
California sea lion	10	1.0442	239.2301	0.5342	0.8972	8.6	0
California sea lion	10	0.6015	176.5835	0.6004	-0.0358	1.8	1
California sea lion	10	0.5022	196.1744	0.4823	0.1399	29.1	A
California sea lion	10	0.9134	107.7399	0.2783	-0.87	20.4	1
California sea lion	10	0.9203	75.589	-0.229	-0.8914	21.1	B
California sea lion	10	1.151	13.9133	-1.1172	-0.2768	2.5	A
California sea lion	10	0.4494	25.0778	-0.407	-0.1905	12.8	0
California sea lion	10	1.5588	31.0625	-1.3352	-0.8043	16.6	1
California sea lion	10	0.7987	9.7276	-0.7873	-0.135	4.5	3
California sea lion	10	0.232	56.0575	-0.1295	-0.1924	1.2	A
California sea lion	10	2.4195	127.0849	1.4592	-1.9302	14.3	2
California sea lion	10	2.5657	180.8686	2.5654	0.0389	0.3	B
California sea lion	10	0.7992	106.8396	0.2316	-0.765	10	3
California sea lion	10	1.027	0.6587	-1.0269	-0.0118	10.3	B
California sea lion	10	1.118	183.6582	1.1157	0.0713	20.1	A
California sea lion	10	2.8924	52.5028	-1.7604	-2.2948	1.4	B

California sea lion	10	0.461	260.5476	0.0757	0.4547	5.6	2
California sea lion	10	0.4809	236.3903	0.2662	0.4005	17.1	1
California sea lion	10	0.7281	294.5816	-0.3029	0.6621	0.2	A
California sea lion	10	0.6091	337.444	-0.5625	0.2337	24.2	0
California sea lion	10	3.8397	135.1914	2.7246	-2.706	12	A
California sea lion	10	0.3867	296.4387	-0.1722	0.3462	0.3	A
California sea lion	10	0.2719	312.357	-0.1832	0.2009	27.9	0
California sea lion	10	1.326	113.0194	0.5186	-1.2204	7.4	2
California sea lion	10	24.1663	107.6507	7.3577	-23.0287	2.6	A
California sea lion	10	2.0479	252.601	0.6126	1.9542	16.3	1
California sea lion	10	2.2444	19.3379	-2.1177	-0.7432	18.8	B
California sea lion	10	7.4356	292.8597	-2.8862	6.8516	18.4	B
California sea lion	10	4.8963	21.934	-4.5417	-1.829	25.7	A
California sea lion	10	0.1284	287.2157	-0.038	0.1226	25.4	1
California sea lion	10	0.9734	103.0884	0.2205	-0.9482	17.8	1
California sea lion	10	5.5724	75.1664	-1.4251	-5.3867	12.8	0
California sea lion	10	49.2715	287.0803	-14.3603	47.0985	3.8	B
California sea lion	10	0.9843	286.1478	-0.2737	0.9454	0.1	1
California sea lion	10	7.9963	91.9549	0.276	-7.9916	24.7	A
California sea lion	10	0.7072	83.4275	-0.0809	-0.7026	25.8	1
California sea lion	10	1.1428	36.683	-0.9164	-0.6827	19.4	B
California sea lion	10	3.4075	73.9903	-0.9392	-3.2753	0.4	2
California sea lion	10	0.6251	355.2511	-0.623	0.0518	24.9	B
California sea lion	10	1.7735	109.2577	0.5851	-1.6743	12	0
California sea lion	10	1.243	321.8757	-0.9778	0.7674	16.6	1
California sea lion	10	2.8358	259.8536	0.5	2.7914	13.8	B
California sea lion	10	0.5025	196.7119	0.4812	0.1445	3.6	B
California sea lion	10	4.1571	340.2654	-3.9128	1.4037	4.1	A
California sea lion	11	0.486	112.3665	0.185	-0.4495	14.7	1
California sea lion	11	6.5284	86.0718	-0.4449	-6.5131	12.9	2
California sea lion	11	2.1271	13.7548	-2.0661	-0.5058	6.7	0
California sea lion	11	2.2553	46.8636	-1.5419	-1.6458	15.7	A
California sea lion	11	0.604	208.5081	0.5308	0.2883	5.2	A
California sea lion	11	3.4213	192.9679	3.3341	0.7678	7.5	0
California sea lion	11	0.4881	275.0358	-0.0428	0.4862	11.9	1
California sea lion	11	4.7465	134.3068	3.316	-3.3966	7.8	B
California sea lion	11	2.8177	94.7776	0.2351	-2.8079	14.4	B
California sea lion	11	2.0991	262.454	0.2759	2.081	6.6	0
California sea lion	11	5.4293	154.4383	4.8982	-2.3426	4.8	0
California sea lion	11	7.3846	286.4354	-2.0868	7.0828	21.8	0
California sea lion	11	1.7016	143.6057	1.3697	-1.0096	24.9	B
California sea lion	11	0.8169	238.4979	0.4269	0.6965	19	1
California sea lion	11	1.0005	138.253	0.7465	-0.6662	15.6	0
California sea lion	11	17.185	106.8491	4.995	-16.4473	22.1	0
California sea lion	11	1.1892	145.1308	0.9757	-0.6799	17.4	1
California sea lion	11	3.3482	264.2895	0.3337	3.3315	13.1	0
California sea lion	11	0.2617	88.9541	-0.0048	-0.2616	18.6	1
California sea lion	11	1.4553	74.3921	-0.3914	-1.4016	4.2	0
California sea lion	11	7.8658	179.6108	7.8657	-0.0534	13.6	A
California sea lion	11	3.1055	283.4624	-0.7225	3.0201	2.7	0
California sea lion	11	1.1479	130.8194	0.7504	-0.8687	10.7	B
California sea lion	11	10.8543	62.0351	-5.0851	-9.5869	3.7	B

California sea lion	11	21.1081	257.751	4.5008	20.6276	24.9	A
California sea lion	11	1.0912	98.2769	0.1572	-1.0799	35.5	1
California sea lion	11	0.4057	12.0862	-0.3967	-0.0849	9.3	0
California sea lion	11	1.1379	304.3018	-0.6412	0.94	20	B
California sea lion	11	1.0687	75.5342	-0.2669	-1.0349	9.8	1
California sea lion	11	2.1886	234.5007	1.271	1.7817	23.5	0
California sea lion	11	0.807	352.0166	-0.7992	0.1121	11.6	1
California sea lion	11	0.8484	320.8933	-0.6583	0.5351	10	1
California sea lion	11	0.6443	316.7879	-0.4696	0.4411	1.9	2
California sea lion	11	9.9292	272.2504	-0.3847	9.9216	14.7	B
California sea lion	11	1.1467	82.5211	-0.1492	-1.137	11.9	1
California sea lion	11	3.9763	182.3749	3.9729	0.1648	0.7	0
California sea lion	11	0.787	186.37	0.7822	0.0873	1.4	1
California sea lion	11	1.7003	241.4652	0.8123	1.4938	0.4	B
California sea lion	11	10.2766	104.6431	2.6031	-9.9429	18.4	B
California sea lion	11	0.7213	71.4027	-0.23	-0.6837	0.3	1
California sea lion	11	1.0022	216.9189	0.8013	0.602	19	1
California sea lion	11	1.7375	257.1107	0.3877	1.6937	10.4	0
California sea lion	11	0.776	69.1491	-0.2762	-0.7252	6.2	1
California sea lion	11	1.4303	76.6866	-0.3293	-1.3919	10.4	1
California sea lion	11	0.4052	227.8789	0.2717	0.3005	12.1	A
California sea lion	11	0.6841	243.9201	0.3008	0.6145	13.6	1
California sea lion	11	1.4471	67.2083	-0.5605	-1.3341	7.2	B
California sea lion	11	39.0777	255.9297	9.5767	37.9054	13.8	B
California sea lion	11	5.7815	75.589	-1.4372	-5.5996	8.2	B
California sea lion	11	117.5395	222.8914	86.459	79.997	0.5	Z
California sea lion	11	0.9358	302.9271	-0.5086	0.7855	15.5	0
California sea lion	11	0.2904	11.2282	-0.2848	-0.0565	7.1	0
California sea lion	11	2.5521	120.6384	1.3008	-2.1958	16.3	A
California sea lion	11	32.702	74.1383	-8.8871	-31.4569	13.5	0
California sea lion	11	0.0905	115.5092	0.039	-0.0816	5.4	3
California sea lion	11	10.5005	300.8781	-5.3847	9.0122	20.9	B
California sea lion	11	5.081	146.1275	4.2191	-2.8319	8.2	A
California sea lion	11	0.3536	98.9142	0.0548	-0.3493	13.7	3
California sea lion	11	4.4034	76.498	-1.0271	-4.2817	11.3	B
California sea lion	11	7.0038	107.2729	2.082	-6.688	20.6	0
California sea lion	11	1.9051	48.0446	-1.2735	-1.4167	6.8	2
California sea lion	12	1.6979	226.5978	1.1667	1.2336	27.5	1
California sea lion	12	7.0844	43.0184	-5.1784	-4.8332	0.3	A
California sea lion	12	8.4377	249.4088	2.9708	7.8987	10	A
California sea lion	12	1.2714	46.4092	-0.8766	-0.9209	0.3	B
California sea lion	12	0.5561	125.4598	0.3226	-0.4529	8.5	1
California sea lion	12	10.7346	76.5982	-2.4824	-10.4422	0.1	0
California sea lion	12	10.8744	153.5589	9.738	-4.8421	0.2	B
California sea lion	12	0.7199	65.817	-0.2949	-0.6567	3.7	0
California sea lion	12	1.5036	306.6599	-0.8977	1.2062	0.9	A
California sea lion	12	1.6732	113.4429	0.6658	-1.5351	23	1
California sea lion	12	0.5954	52.1541	-0.3653	-0.4702	0.1	1
California sea lion	12	0.4178	98.6801	0.0631	-0.413	0.3	2
California sea lion	12	0.7815	344.6114	-0.7534	0.2074	0.5	1
California sea lion	12	0.5501	331.7837	-0.4847	0.2601	10	1
California sea lion	12	0.2438	287.3381	-0.0727	0.2328	0.4	1

California sea lion	12	4.6705	78.1937	-0.9545	-4.5717	0.4	0
California sea lion	12	0.9856	259.2062	0.1846	0.9682	1.6	1
California sea lion	12	3.4003	2.5782	-3.3969	-0.153	0.3	2
California sea lion	12	1.0601	259.3835	0.1954	1.0419	10.5	2
California sea lion	12	0.6543	287.4479	-0.1962	0.6242	3.8	1
California sea lion	12	0.8686	86.5822	-0.0517	-0.867	12.1	1
California sea lion	12	0.6531	89.3032	-0.0079	-0.6531	1.3	1
California sea lion	12	7.6968	121.2036	3.9898	-6.5833	0.1	A
California sea lion	12	9.1779	276.3915	-1.0174	9.1209	14.4	0
California sea lion	12	0.2417	84.1298	-0.0247	-0.2404	1	1
California sea lion	12	0.6485	281.9992	-0.1348	0.6343	4.9	1
California sea lion	12	1.6946	114.581	0.7051	-1.5411	0.3	B
California sea lion	12	0.3015	333.3251	-0.2694	0.1354	13.5	2
California sea lion	12	1.8494	179.4411	1.8493	-0.018	0.2	A
California sea lion	12	1.412	16.981	-1.3504	-0.4124	0.6	1
California sea lion	12	0.8652	270.0872	-0.0013	0.8652	16.2	1
California sea lion	12	11.9257	79.065	-2.2552	-11.7092	0.3	B
California sea lion	12	1.6115	99.1805	0.2572	-1.5909	4.5	A
California sea lion	12	15.0174	335.9202	-13.7086	6.1272	15.4	A
California sea lion	12	1.441	248.1939	0.5354	1.3379	10.8	0
California sea lion	12	0.4469	315.0556	-0.3163	0.3157	30.9	0
California sea lion	12	0.6288	91.4599	0.016	-0.6286	14.3	3
California sea lion	12	0.9601	18.2827	-0.9116	-0.3012	13	1
California sea lion	12	1.2964	143.2808	1.0392	-0.7751	0.8	1
California sea lion	12	0.3191	46.0759	-0.2214	-0.2299	5.2	1
California sea lion	12	0.6227	60.9076	-0.3028	-0.5442	5.2	0
California sea lion	12	0.6761	261.1959	0.1035	0.6681	3.8	2
California sea lion	12	1.0699	88.1017	-0.0354	-1.0693	0.4	1
California sea lion	12	4.818	293.575	-1.9259	4.4158	1.5	A
California sea lion	12	0.3422	216.6022	0.2747	0.204	2.2	1
California sea lion	12	16.2668	73.3729	-4.6423	-15.5867	11	B
California sea lion	12	1.9835	67.1211	-0.771	-1.8275	18.6	1
California sea lion	12	1.141	281.3351	-0.2242	1.1188	15.1	2
California sea lion	13	2.17	147.7924	1.8362	-1.1566	1.5	0
California sea lion	13	1.215	202.9444	1.1189	0.4737	12.7	1
California sea lion	13	0.4084	72.9479	-0.1198	-0.3905	5.2	2
California sea lion	13	0.3221	66.3589	-0.1291	-0.295	13.3	1
California sea lion	13	0.5144	102.021	0.1072	-0.5031	8.6	1
California sea lion	13	0.2901	34.8126	-0.2382	-0.1656	13.7	1
California sea lion	13	1.2664	9.867	-1.2477	-0.217	23.1	2
California sea lion	13	1.0544	141.1808	0.8215	-0.661	8	1
California sea lion	13	90.9164	323.8458	-73.2594	53.6361	31.7	Z
California sea lion	13	0.2039	161.0328	0.1928	-0.0663	11	2
California sea lion	13	8.768	32.3563	-7.4055	-4.6925	7.9	A
California sea lion	13	0.0858	313.5291	-0.0591	0.0622	17	2
California sea lion	13	8.3674	20.4751	-7.8383	-2.9269	24.7	A
California sea lion	13	1.5987	293.4612	-0.6364	1.4665	10.4	0
California sea lion	13	0.5876	200.1791	0.5515	0.2027	0.5	2
California sea lion	13	0.6384	168.2137	0.625	-0.1304	10.1	A
California sea lion	13	8.7074	132.7628	5.9142	-6.3927	14.4	B
California sea lion	13	7.3637	107.9474	2.2716	-7.0053	15.1	B
California sea lion	13	0.7959	262.526	0.1036	0.7891	26.6	1

California sea lion	13	0.4672	275.3704	-0.0437	0.4652	8.2	1
California sea lion	13	0.4269	39.3275	-0.3302	-0.2706	18	1
California sea lion	13	0.2543	185.6187	0.253	0.0249	17.1	2
California sea lion	13	0.2097	113.6526	0.0841	-0.1921	18.8	1
California sea lion	13	2.6251	98.1731	0.3735	-2.5984	15.8	1
California sea lion	13	0.3522	241.9531	0.1656	0.3108	6.6	2
California sea lion	13	0.4988	59.7923	-0.251	-0.4311	13.6	2
California sea lion	13	0.7317	52.9304	-0.4411	-0.5839	1	1
California sea lion	13	0.7778	77.2108	-0.1722	-0.7585	11.8	2
California sea lion	13	0.7783	106.2639	0.218	-0.7472	8.3	2
California sea lion	13	0.9642	269.2257	0.0131	0.9642	10.3	1
California sea lion	13	2.8915	80.8248	-0.4606	-2.8545	12.4	0
California sea lion	13	2.5456	251.8471	0.7934	2.4189	5.9	B
California sea lion	13	1.0085	249.7261	0.3495	0.9461	16.6	2
California sea lion	13	9.2206	280.0328	-1.602	9.0796	0.5	0
California sea lion	13	3.7574	48.7544	-2.4768	-2.8251	10.1	A
California sea lion	13	0.6777	73.1103	-0.1969	-0.6485	8.2	2
California sea lion	13	2.9487	77.4518	-0.6402	-2.8783	14.5	0
California sea lion	13	2.1341	248.8832	0.769	1.9908	16.7	2
California sea lion	13	1.0906	62.2006	-0.5086	-0.9647	6.7	1
California sea lion	13	0.4987	235.0878	0.2854	0.4089	6.7	1
California sea lion	13	0.7734	15.3907	-0.7457	-0.2053	15.8	1
California sea lion	13	4.5041	325.8402	-3.7267	2.5291	8.4	0
California sea lion	13	2.6086	176.8164	2.6046	-0.1449	24.1	1
California sea lion	13	0.2539	143.7348	0.2047	-0.1502	15	A
California sea lion	13	0.7612	86.9508	-0.0405	-0.7601	3.3	1
California sea lion	13	1.4935	254.9255	0.3885	1.4421	12.9	1
California sea lion	13	0.7893	70.5259	-0.2631	-0.7441	3.2	2
California sea lion	13	2.4073	246.9113	0.9443	2.2145	10.7	A
California sea lion	13	1.6412	189.2872	1.6197	0.2649	7.7	1
California sea lion	13	1.0795	225.671	0.7544	0.7722	10.2	2
California sea lion	13	0.6323	101.9394	0.1308	-0.6187	5.1	1
California sea lion	13	1.7926	64.7108	-0.7656	-1.6208	17.6	1
California sea lion	13	0.6195	225.2174	0.4364	0.4397	9.8	1
California sea lion	13	4.9654	312.2685	-3.3391	3.6744	5.6	A
California sea lion	13	0.2083	292.0427	-0.0782	0.193	19.6	3
California sea lion	13	0.5247	186.1936	0.5216	0.0566	8.7	2
California sea lion	13	6.8943	78.2095	-1.4063	-6.7488	12	B
California sea lion	13	1.6086	40.2956	-1.2269	-1.0403	9.9	B
California sea lion	13	3.4393	296.1729	-1.5165	3.0866	26.8	A
California sea lion	13	5.4527	200.4131	5.1105	1.9018	1	B
California sea lion	13	0.7676	55.3267	-0.4367	-0.6313	7.1	1
California sea lion	13	4.4889	264.093	0.463	4.4651	2.3	B
California sea lion	13	1.4458	190.9998	1.4193	0.2759	10.3	0
California sea lion	13	0.3012	239.2539	0.154	0.2589	6.3	0
California sea lion	13	0.6609	76.4973	-0.1543	-0.6426	17.5	1
California sea lion	13	0.4344	56.6593	-0.2387	-0.3629	12.8	1
California sea lion	13	2.4426	165.9872	2.37	-0.5915	26	0
California sea lion	13	4.3006	354.88	-4.2835	0.3838	0.2	A
California sea lion	13	0.2214	194.7402	0.2141	0.0563	9.9	1
California sea lion	13	8.8934	101.813	1.8246	-8.705	4.7	B
California sea lion	13	2.238	226.2735	1.5471	1.6173	8.1	A

California sea lion	13	2.865	252.4224	0.8656	2.7313	7.2	0
California sea lion	13	9.1407	140.9473	7.1001	-5.759	2.1	1
California sea lion	13	3.2874	357.3992	-3.284	0.1492	15.1	1
California sea lion	13	1.8395	118.721	0.8841	-1.6132	17.9	A
California sea lion	14	2.7264	102.1685	0.575	-2.6651	23.9	1
California sea lion	14	4.8514	250.4177	1.6271	4.5708	13.6	B
California sea lion	14	0.5234	29.3085	-0.4564	-0.2562	21.2	2
California sea lion	14	1.9162	241.0302	0.9283	1.6764	12.9	A
California sea lion	14	1.7472	251.1934	0.5634	1.6539	1.3	A
California sea lion	14	3.7843	180.1107	3.7843	0.0073	0.5	A
California sea lion	14	1.4391	66.7391	-0.5682	-1.3221	18.7	1
California sea lion	14	1.6215	261.6957	0.2343	1.6045	7.8	1
California sea lion	14	3.927	175.2898	3.9137	-0.3225	17.7	0
California sea lion	14	1.0658	296.7485	-0.4796	0.9517	5.4	1
California sea lion	14	1.3479	138.2522	1.0057	-0.8975	20.2	1
California sea lion	14	0.653	321.1626	-0.5086	0.4095	15.4	A
California sea lion	14	6.792	281.7335	-1.3789	6.6501	0.2	0
California sea lion	14	1.2369	282.0881	-0.259	1.2095	8.2	A
California sea lion	14	0.5514	292.9584	-0.2151	0.5077	8.5	1
California sea lion	14	0.5563	105.6992	0.1505	-0.5356	3.9	2
California sea lion	14	0.9107	146.4664	0.7592	-0.5031	5.2	A
California sea lion	14	0.9306	301.9623	-0.4926	0.7895	1.6	B
California sea lion	14	0.7815	263.4149	0.0897	0.7763	0.9	A
California sea lion	14	21.5994	310.5747	-14.0351	16.406	19.9	B
California sea lion	14	29.2384	236.3151	16.2476	24.3293	22.4	B
California sea lion	14	1.6734	16.8444	-1.6016	-0.4849	18.3	B
California sea lion	14	5.0908	111.6029	1.8755	-4.7332	0.2	0
California sea lion	14	0.2172	95.5473	0.021	-0.2161	7.2	2
California sea lion	14	0.4633	68.3839	-0.1707	-0.4307	15	A
California sea lion	14	0.2973	23.5839	-0.2725	-0.119	4.9	1
California sea lion	14	0.2864	3.0683	-0.2859	-0.0153	9.4	1
California sea lion	14	0.1127	202.6322	0.104	0.0433	18	2
California sea lion	14	1.388	114.4266	0.574	-1.2637	11.8	1
California sea lion	14	0.2425	166.9186	0.2362	-0.0549	30.2	1
California sea lion	14	1.2274	208.1169	1.0825	0.5784	0.3	1
California sea lion	14	2.1087	291.0032	-0.7556	1.9686	13.7	A
California sea lion	14	0.4366	346.3652	-0.4243	0.1029	13.7	A
California sea lion	14	0.4893	298.8422	-0.236	0.4286	5.1	1
California sea lion	15	0.8308	295.513	-0.3578	0.7498	9.8	1
California sea lion	15	1.3276	185.2217	1.3221	0.1208	2.5	A
California sea lion	15	2.1904	248.8923	0.789	2.0434	0.4	3
California sea lion	15	6.5347	164.8521	6.3078	-1.7076	16.1	B
California sea lion	15	0.9818	142.9744	0.7839	-0.5912	15.1	A
California sea lion	15	1.0133	303.5611	-0.5601	0.8444	4	A
California sea lion	15	0.7772	235.5747	0.4394	0.6411	4.6	1
California sea lion	15	0.8235	13.0394	-0.8023	-0.1858	9.1	B
California sea lion	15	0.2493	212.1007	0.2112	0.1325	11.6	3
California sea lion	15	1.3676	35.6851	-1.1108	-0.7978	26.1	A
California sea lion	15	4.7979	109.9299	1.6365	-4.5106	14.5	B
California sea lion	15	0.9785	216.6041	0.7856	0.5835	4.9	1
California sea lion	15	0.881	279.3925	-0.1437	0.8692	1.5	1
California sea lion	15	14.121	42.1688	-10.4613	-9.4796	9.9	B

California sea lion	15	8.5616	257.9235	1.7949	8.3721	19.2	0
California sea lion	15	0.5225	199.4718	0.4926	0.1742	10.9	2
California sea lion	15	0.5119	319.6877	-0.3903	0.3312	11.1	3
California sea lion	15	0.3303	215.4454	0.2691	0.1916	16.5	1
California sea lion	15	3.0474	352.6741	-3.0225	0.3886	17.1	2
California sea lion	15	0.8766	37.9955	-0.6908	-0.5396	31.5	0
California sea lion	15	2.3643	172.838	2.3458	-0.2948	4	A
California sea lion	15	0.894	256.8244	0.2038	0.8705	14.6	B
California sea lion	15	2.9127	183.1908	2.9082	0.1621	26.3	1
California sea lion	15	0.7759	158.9451	0.7241	-0.2787	9.7	B
California sea lion	15	38.4037	283.9532	-9.1872	37.2705	7.5	0
California sea lion	15	0.4593	111.9535	0.1717	-0.426	15.9	2
California sea lion	15	0.478	243.9208	0.2101	0.4293	16.4	1
California sea lion	15	0.716	124.1405	0.4018	-0.5926	4.6	A
California sea lion	15	0.1947	342.5445	-0.1857	0.0584	12.4	1
California sea lion	15	1.2451	163.8644	1.1961	-0.346	10.5	2
California sea lion	15	6.3218	71.0861	-2.0473	-5.9805	9.1	A
California sea lion	15	0.3397	251.4946	0.1078	0.3222	0.7	1
California sea lion	15	1.3331	137.8602	0.9885	-0.8944	1.4	B
California sea lion	15	0.2336	345.2004	-0.2259	0.0597	19.3	3
California sea lion	15	3.6138	65.713	-1.4858	-3.294	5.6	B

Species	Animal ID	error (km)	angle	lat error(km)	lon error (km)	GPS residual	ARGOS LC
Cape fur seal	1	3.14	157.4799	2.9005	-1.2027	3.3	B
Cape fur seal	1	0.7878	88.2139	-0.0246	-0.7874	0.7	1
Cape fur seal	1	8.5366	280.6504	-1.5808	8.3895	8.1	0
Cape fur seal	1	2.4321	298.0294	-1.1431	2.1468	8.6	0
Cape fur seal	1	5.4206	144.6136	4.4188	-3.139	3.1	A
Cape fur seal	1	0.5968	300.2352	-0.3005	0.5156	0.1	1
Cape fur seal	1	4.131	220.5046	3.1407	2.6831	0.2	A
Cape fur seal	1	1.3423	140.4835	1.0355	-0.8541	5	1
Cape fur seal	1	0.6633	307.1165	-0.4003	0.529	0.1	1
Cape fur seal	1	1.4959	48.2782	-0.9956	-1.1165	0.3	Z
Cape fur seal	1	1.3072	22.3547	-1.209	-0.4972	0.9	1
Cape fur seal	1	6.4535	170.9092	6.3724	-1.0197	11.7	A
Cape fur seal	1	0.705	76.9428	-0.1593	-0.6868	2.2	2
Cape fur seal	1	0.2931	293.3966	-0.1164	0.269	0.5	0
Cape fur seal	1	1.7655	93.2457	0.0998	-1.7627	7	A
Cape fur seal	1	1.4837	68.1829	-0.5515	-1.3774	10.7	2
Cape fur seal	1	0.4329	201.1612	0.4037	0.1563	0.2	1
Cape fur seal	1	3.3707	18.765	-3.1915	-1.0843	21.6	1
Cape fur seal	1	5.8345	348.5031	-5.7175	1.1629	11.7	0
Cape fur seal	1	1.008	292.3641	-0.3836	0.9322	0.2	1
Cape fur seal	1	9.3958	38.4629	-7.3585	-5.8442	0.2	A
Cape fur seal	1	4.5922	248.0727	1.7141	4.26	10.3	A
Cape fur seal	1	0.9152	253.9745	0.2526	0.8796	8.1	A
Cape fur seal	2	1.234	348.8371	-1.2106	0.2389	1.2	A
Cape fur seal	2	1.1919	125.0391	0.6843	-0.9759	7.9	0
Cape fur seal	2	7.0658	86.8014	-0.3965	-7.0548	3.7	0
Cape fur seal	2	0.4394	325.003	-0.3599	0.252	0.2	2
Cape fur seal	2	6.1052	186.1915	6.0696	0.6585	29.6	A
Cape fur seal	2	6.2893	4.5349	-6.2697	-0.4973	0.1	A
Cape fur seal	2	2.1953	232.3435	1.341	1.738	0.3	A
Cape fur seal	2	0.2654	169.7741	0.2611	-0.0471	1.2	2
Cape fur seal	2	1.0961	12.838	-1.0687	-0.2436	8.4	A
Cape fur seal	2	2.5258	47.0163	-1.7222	-1.8478	14.4	3
Cape fur seal	2	1.9097	76.0118	-0.4618	-1.8531	0.2	B
Cape fur seal	2	0.5511	185.0022	0.549	0.0481	3.3	A
Cape fur seal	2	5.7273	336.9131	-5.2688	2.2458	6.5	A
Cape fur seal	2	12.3788	293.1618	-4.8747	11.381	6.9	B
Cape fur seal	2	1.8095	172.8727	1.7955	-0.2245	9	A
Cape fur seal	2	7.9015	73.981	-2.1831	-7.5947	8.2	0
Cape fur seal	2	0.4661	319.8506	-0.3563	0.3005	13.5	1
Cape fur seal	2	1.7741	159.2852	1.6594	-0.6275	0.6	B
Cape fur seal	2	2.7076	224.245	1.9395	1.8892	1.3	A
Cape fur seal	2	0.2372	348.7276	-0.2326	0.0464	4.2	3
Cape fur seal	2	22.5249	264.7083	2.0549	22.4289	23.5	A
Cape fur seal	2	4.6957	267.1074	0.236	4.6898	17.3	A
Cape fur seal	2	0.2944	145.2391	0.2419	-0.1678	4.4	0
Cape fur seal	2	0.4084	298.1998	-0.193	0.36	4.6	A
Cape fur seal	2	0.9616	119.3961	0.4719	-0.8378	0.3	B
Cape fur seal	2	4.411	280.1604	-0.779	4.3418	6	B
Cape fur seal	2	0.9328	13.6966	-0.9063	-0.2209	0.3	B
Cape fur seal	2	2.7269	103.0327	0.6146	-2.6566	11.9	B
Cape fur seal	2	8.695	257.447	1.8866	8.4872	0.1	A

Cape fur seal	2	48.8178	310.535	-31.7888	37.1019	6.5	A
Cape fur seal	2	3.0169	248.5782	1.1015	2.8085	0.3	0
Cape fur seal	3	9.3812	264.4822	0.8982	9.3378	5.9	A
Cape fur seal	3	1.4758	39.4257	-1.14	-0.9373	18.4	0
Cape fur seal	3	0.185	236.6462	0.1017	0.1545	18.4	1
Cape fur seal	3	46.3968	53.5114	-27.6531	-37.3018	4	B
Cape fur seal	3	19.2089	114.6403	7.9948	-17.4598	0.5	A
Cape fur seal	3	5.1463	17.8598	-4.8984	-1.5783	0.3	0
Cape fur seal	3	0.6908	3.2235	-0.6897	-0.0388	24.7	0
Cape fur seal	3	16.5322	199.1439	15.6166	5.4216	2.4	A
Cape fur seal	3	4.2767	60.5166	-2.1055	-3.7228	6.7	A
Cape fur seal	3	4.8131	198.1847	4.5726	1.5021	10.1	B
Cape fur seal	3	2.4875	127.3013	1.5073	-1.9787	9.7	B
Cape fur seal	3	1.1119	203.1106	1.0227	0.4364	0.1	B
Cape fur seal	3	0.554	160.9731	0.5237	-0.1806	13.3	1
Cape fur seal	3	0.3356	9.2937	-0.3312	-0.0542	22.7	1
Cape fur seal	3	0.7539	208.3354	0.6636	0.3578	21.3	A
Cape fur seal	3	1.3666	158.0762	1.2678	-0.5103	3.4	1
Cape fur seal	3	1.2506	98.0513	0.1751	-1.2383	11.4	1
Cape fur seal	3	3.229	342.0034	-3.0711	0.9976	23	B
Cape fur seal	3	1.2418	50.5876	-0.7884	-0.9594	4.2	0
Cape fur seal	3	2.3942	286.8011	-0.6923	2.292	18.1	A
Cape fur seal	3	0.2109	49.2861	-0.1376	-0.1599	13.9	1
Cape fur seal	3	2.5128	351.8558	-2.4875	0.356	1	0
Cape fur seal	4	3.4261	236.8089	1.8752	2.8671	0.1	0
Cape fur seal	4	4.4337	62.5663	-2.0434	-3.9351	0.3	A
Cape fur seal	4	3.1144	173.8168	3.0963	-0.3354	9	B
Cape fur seal	4	1.9899	305.1554	-1.1459	1.627	10	B
Cape fur seal	4	1.3998	245.7981	0.5738	1.2767	16.5	B
Cape fur seal	4	4.3753	78.05	-0.9069	-4.2804	12.1	B
Cape fur seal	4	22.2144	337.8351	-20.5765	8.3809	22.3	B
Cape fur seal	4	3.9506	328.9451	-3.3846	2.038	0.7	1
Cape fur seal	4	1.5828	65.7169	-0.651	-1.4428	1.5	0
Cape fur seal	4	6.4431	74.1377	-1.7631	-6.1978	0.3	B
Cape fur seal	5	1.0076	273.432	-0.0604	1.0058	12.4	1
Cape fur seal	5	1.6325	160.0608	1.5346	-0.5567	26.1	1
Cape fur seal	5	1.9855	298.2723	-0.9406	1.7486	26.6	0
Cape fur seal	5	11.2254	213.2542	9.3855	6.1555	7.9	B
Cape fur seal	5	1.0569	277.5238	-0.1384	1.0478	13.3	1
Cape fur seal	5	1.8268	120.8194	0.9358	-1.5688	12.3	A
Cape fur seal	5	2.9128	264.9535	0.2559	2.9015	9	A
Cape fur seal	5	2.4861	118.5816	1.1892	-2.1831	14.1	2
Cape fur seal	5	4.3179	74.9314	-1.1233	-4.1694	6.5	0
Cape fur seal	5	2.9437	65.8412	-1.2051	-2.6859	7.4	A
Cape fur seal	5	14.1382	151.2906	12.3982	-6.7915	7.4	A
Cape fur seal	5	2.6956	256.4707	0.6303	2.6208	17.3	0
Cape fur seal	5	1.4329	291.3983	-0.5229	1.3342	8.8	B
Cape fur seal	5	0.4751	281.3087	-0.0932	0.4659	3.5	0
Cape fur seal	5	2.472	128.0021	1.5218	-1.9479	14	A
Cape fur seal	5	9.3748	242.9667	4.2579	8.3505	6.5	B
Cape fur seal	5	1.5576	294.8177	-0.6539	1.4137	18.9	0
Cape fur seal	6	8.7726	109.8055	2.9694	-8.2537	1	B
Cape fur seal	6	11.0637	297.4973	-5.1125	9.8139	11.8	B

Cape fur seal	6	6.449	303.1168	-3.5247	5.4014	3.5	A
Cape fur seal	6	2.3974	182.9392	2.3943	0.1229	20.3	A
Cape fur seal	6	10.8936	85.2715	-0.9034	-10.8565	11.3	A
Cape fur seal	6	12.2416	62.2456	-5.706	-10.8332	17.1	B
Cape fur seal	6	1.8897	13.4333	-1.838	-0.439	3.1	A
Cape fur seal	6	4.8962	152.8537	4.3566	-2.2339	13.1	B
Cape fur seal	6	1.597	141.5856	1.2513	-0.9923	0.2	A
Cape fur seal	6	3.6245	77.8	-0.7665	-3.5426	1.8	B

Species	Animal ID	error (km)	angle	lat error(km)	lon error (km)	GPS residual	ARGOS LC
Australian fur seal	16	0.2627	235.1862	0.15	0.2156	28.9396	0
Australian fur seal	16	0.9053	149.0663	0.7765	-0.4654	11.2325	1
Australian fur seal	16	0.8905	276.2782	-0.0974	0.8852	15.5906	2
Australian fur seal	16	0.418	227.4314	0.2827	0.3078	2.8841	2
Australian fur seal	16	0.4122	290.8942	-0.147	0.3851	8.5697	2
Australian fur seal	16	17.733	98.26	2.5279	-17.549	20.49	0
Australian fur seal	16	1.4563	91.9506	0.0494	-1.4554	6.8901	1
Australian fur seal	16	4.4292	238.5977	2.3069	3.7805	4.363	0
Australian fur seal	16	2.1533	249.7061	0.7466	2.0197	11.1215	1
Australian fur seal	16	0.0598	65.3528	-0.0249	-0.0544	10.7304	3
Australian fur seal	16	5.0494	248.4609	1.8524	4.6968	21.605	B
Australian fur seal	16	2.4138	89.1	-0.0383	-2.4135	0.1737	0
Australian fur seal	16	14.3929	126.8312	8.6195	-11.5201	24.3382	B
Australian fur seal	16	0.2165	107.2228	0.0641	-0.2068	5.7332	3
Australian fur seal	16	0.5995	116.1496	0.2642	-0.5381	1.8594	1
Australian fur seal	16	2.6503	124.5208	1.5016	-2.1836	10.7111	1
Australian fur seal	16	1.0736	251.0237	0.349	1.0152	10.228	2
Australian fur seal	16	0.2742	343.8111	-0.2634	0.0765	14.8279	2
Australian fur seal	16	1.2138	327.3651	-1.0222	0.6546	19.0655	A
Australian fur seal	16	8.6366	222.8094	6.3338	5.8691	19.8372	A
Australian fur seal	16	0.2478	87.3898	-0.0113	-0.2475	4.3185	2
Australian fur seal	16	0.5343	238.3987	0.28	0.4551	0.325	1
Australian fur seal	16	3.8939	256.0069	0.9407	3.7784	2.1464	0
Australian fur seal	16	2.9895	22.688	-2.7583	-1.1531	6.9383	1
Australian fur seal	16	71.7149	93.7709	4.3823	-71.5604	3.4783	B
Australian fur seal	17	1.9301	66.5967	-0.7668	-1.7713	0	1
Australian fur seal	17	0.5486	77.572	-0.1181	-0.5357	0	3
Australian fur seal	17	0.5009	274.8737	-0.0426	0.4991	0	3
Australian fur seal	18	3.896	112.3923	1.4833	-3.6022	8.9237	1
Australian fur seal	18	1.5084	268.8666	0.0297	1.5081	12.6886	2
Australian fur seal	18	9.3316	76.2993	-2.2155	-9.0661	20.5497	0
Australian fur seal	18	1.8911	299.474	-0.9307	1.6464	8.9137	A
Australian fur seal	18	8.2771	112.8281	3.2075	-7.6288	11.6571	0
Australian fur seal	18	11.494	260.5524	1.8785	11.3381	11.1548	0
Australian fur seal	18	5.0423	200.8249	4.7127	1.7926	14.0446	1
Australian fur seal	18	9.7135	56.5953	-5.3521	-8.1089	19.5587	Z
Australian fur seal	18	83.8771	325.7124	-69.4482	47.2513	24.2711	A
Australian fur seal	18	51.2637	78.6005	-10.2977	-50.2527	13.4405	0
Australian fur seal	18	184.2988	249.0187	64.0547	172.0873	17.9864	B
Australian fur seal	18	140.9606	89.9161	-1.5209	-140.9666	1.086	B
Australian fur seal	18	93.1562	80.0068	-16.7232	-91.7445	14.8686	B
Australian fur seal	18	380.4179	286.1077	-114.4599	365.5725	13.2337	B
Australian fur seal	18	9.5602	152.9553	8.5135	-4.3469	0.424	0
Australian fur seal	18	7.4307	162.4409	7.0842	-2.2418	17.2804	B
Australian fur seal	18	1.8023	70.7538	-0.5943	-1.7016	25.0275	1
Australian fur seal	18	1.5286	118.694	0.7338	-1.3409	11.4812	2
Australian fur seal	18	0.3863	275.2443	-0.0353	0.3847	13.0231	3
Australian fur seal	18	0.4113	87.3873	-0.0188	-0.4108	11.9097	2
Australian fur seal	18	2.4194	293.0205	-0.9465	2.2268	21.8479	0
Australian fur seal	18	8.4813	94.9878	0.7327	-8.4492	5.8446	0
Australian fur seal	18	3.157	129.9376	2.0263	-2.4206	9.089	A
Australian fur seal	18	5.7854	96.4015	0.6429	-5.7493	15.4482	0
Australian fur seal	18	26.7295	264.5954	2.4712	26.6108	0.153	B
Australian fur seal	18	52.9087	241.2186	25.3326	46.3727	20.1742	B

Species	Animal ID	error (km)	angle	lat error(km)	lon error (km)	GPS residual	ARGOS LC
Northern elephant seal	23	0.7045	18.5641	-0.6678	-0.2243	9.6	B
Northern elephant seal	23	4.6766	72.8741	-1.376	-4.4693	13.4	B
Northern elephant seal	23	5.3432	181.5811	5.3412	0.1474	16.5	B
Northern elephant seal	23	6.8157	295.3355	-2.9143	6.1601	17	B
Northern elephant seal	23	15.768	263.2277	1.8742	15.658	9.3	A
Northern elephant seal	23	1.4533	3.2377	-1.451	-0.0821	18	2
Northern elephant seal	23	762.3402	97.6613	137.5762	-756.3309	6.1	Z
Northern elephant seal	23	5.6068	330.087	-4.8594	2.796	14.4	B
Northern elephant seal	23	1.4385	82.8585	-0.1787	-1.4273	3.1	Z
Northern elephant seal	23	9.7141	261.3422	1.4682	9.6034	1.9	B
Northern elephant seal	23	1.0115	84.2131	-0.1019	-1.0064	21.9	B
Northern elephant seal	23	37.7729	268.085	1.3554	37.7519	5.4	A
Northern elephant seal	23	5.7053	141.1762	4.4457	-3.5768	13.9	B
Northern elephant seal	23	2.1134	339.2389	-1.9761	0.7491	19.4	A
Northern elephant seal	23	77.5113	73.9971	-21.0025	-74.5083	13.6	B
Northern elephant seal	23	2.2818	92.6831	0.1072	-2.2793	0.4	0
Northern elephant seal	23	26.1249	283.3707	-5.9983	25.4168	22.5	B
Northern elephant seal	23	70.1035	264.849	6.6201	69.8212	16.7	A
Northern elephant seal	23	1.7898	235.6933	1.0089	1.4785	11.6	0
Northern elephant seal	23	1.0385	197.725	0.9892	0.3162	17.9	B
Northern elephant seal	23	5.2372	213.283	4.3788	2.8741	4.7	B
Northern elephant seal	23	2.1602	184.0435	2.1548	0.1523	4.8	B
Northern elephant seal	23	1.0668	35.1387	-0.8724	-0.614	1.3	B
Northern elephant seal	23	1.0559	158.6084	0.9832	-0.3851	1.8	B
Northern elephant seal	23	105.0672	292.9904	-40.2914	96.7239	0.8	Z
Northern elephant seal	23	1.3446	204.2538	1.226	0.5523	13.4	B
Northern elephant seal	23	24.4576	158.6579	22.7868	-8.901	7.4	A
Northern elephant seal	23	10.3036	25.5531	-9.2941	-4.4444	16.9	B
Northern elephant seal	23	12.4009	240.9659	6.028	10.8425	13.2	A
Northern elephant seal	23	8.7028	333.6952	-7.8004	3.8566	13.6	B
Northern elephant seal	23	15.5538	42.5965	-11.4402	-10.5273	9.5	B
Northern elephant seal	23	1.2478	134.7768	0.879	-0.8858	25.8	B
Northern elephant seal	23	2.8393	119.5447	1.4006	-2.4701	18.3	B
Northern elephant seal	23	0.8281	219.3277	0.6406	0.5248	8.2	B
Northern elephant seal	23	1.8585	106.8715	0.5396	-1.7785	8.3	B
Northern elephant seal	23	4.9422	101.9712	1.0271	-4.8347	10	A
Northern elephant seal	23	1.6866	257.131	0.3759	1.6443	0.7	B
Northern elephant seal	23	3.476	278.5558	-0.5161	3.4373	8.8	B
Northern elephant seal	23	30.2812	104.9873	7.9093	-29.2512	11.7	Z
Northern elephant seal	23	33.6754	252.689	10.1156	32.1502	4.9	A
Northern elephant seal	23	3.0742	58.4226	-1.6092	-2.619	0.2	B
Northern elephant seal	23	5.8484	69.4483	-2.0503	-5.4762	19.1	B
Northern elephant seal	23	30.0925	69.7574	-10.3364	-28.234	12.3	B
Northern elephant seal	23	74.6908	265.4454	6.4621	74.4569	9.7	Z
Northern elephant seal	23	10.6312	90.8781	0.174	-10.6299	18.3	A
Northern elephant seal	23	3.5008	112.3288	1.3311	-3.2383	13.6	B
Northern elephant seal	23	12.7697	236.3324	7.091	10.6278	20.5	B
Northern elephant seal	23	7.5305	287.6833	-2.282	7.1747	11.7	B
Northern elephant seal	23	16.0184	279.4608	-2.6062	15.8006	19.5	A
Northern elephant seal	23	13.4978	302.4468	-7.2282	11.3907	22.4	B
Northern elephant seal	23	814.7328	291.6313	-242.199	759.4014	9.1	B
Northern elephant seal	23	201.0622	297.1495	-88.4813	178.9329	16.4	B
Northern elephant seal	23	2.2321	240.9185	1.0853	1.9507	20	A
Northern elephant seal	23	3.4628	258.9484	0.6649	3.3986	11.2	B
Northern elephant seal	23	1.1989	302.0864	-0.6367	1.0157	18.4	0
Northern elephant seal	23	459.7801	109.0623	168.6872	-434.9116	19.4	Z
Northern elephant seal	23	6.7622	311.5099	-4.4791	5.0638	0.9	A
Northern elephant seal	23	11.5811	319.7848	-8.8383	7.4775	15.5	B
Northern elephant seal	23	5.9725	72.7195	-1.771	-5.7029	8.5	B
Northern elephant seal	23	5.978	67.7681	-2.259	-5.5336	0.9	B
Northern elephant seal	23	13.3066	235.8163	7.4874	11.0077	8.9	B
Northern elephant seal	23	7.4973	260.285	1.2702	7.3897	9.2	B
Northern elephant seal	23	10.9066	292.5625	-4.1755	10.0718	7.4	B
Northern elephant seal	23	1.041	198.622	0.9865	0.3324	18.4	B
Northern elephant seal	23	5.1254	231.2041	3.2128	3.9947	0.4	B
Northern elephant seal	23	6.648	103.44	1.549	-6.4659	1.2	B
Northern elephant seal	23	2.2108	196.2408	2.1226	0.6183	0.6	B

Northern elephant seal	23	12.1585	247.7822	4.6091	11.2558	7	B
Northern elephant seal	23	188.2076	72.8814	-52.5109	-179.8906	0.5	B
Northern elephant seal	23	2.9901	29.2036	-2.6098	-1.4589	0.4	B
Northern elephant seal	23	3.1708	131.2375	2.0907	-2.3844	0.2	B
Northern elephant seal	23	10.2843	237.0086	5.6062	8.626	13.3	B
Northern elephant seal	23	4.0122	317.7392	-2.9688	2.6982	7.8	B
Northern elephant seal	23	19.2955	10.3149	-18.9827	-3.455	16.6	B
Northern elephant seal	23	3.7443	42.9599	-2.7396	-2.5517	8	A
Northern elephant seal	23	1934.0724	97.3722	555.2192	-1940.0932	25.6	A
Northern elephant seal	23	3.819	315.6508	-2.7304	2.6696	14.2	B
Northern elephant seal	23	0.8098	63.2923	-0.3639	-0.7234	15.6	2
Northern elephant seal	23	7.886	149.9879	6.8296	-3.9445	14.4	B
Northern elephant seal	23	5.4569	287.3961	-1.6299	5.2073	0.5	B
Northern elephant seal	23	13.9888	289.3373	-4.6217	13.1996	15.6	B
Northern elephant seal	24	8.1511	111.4372	2.9825	-7.5872	14.5	B
Northern elephant seal	24	3.4066	257.9323	0.7129	3.3313	15.5	B
Northern elephant seal	24	3.9065	124.611	2.2195	-3.2151	5.3	A
Northern elephant seal	24	11.027	292.8967	-4.2841	10.1582	0.8	A
Northern elephant seal	24	377.1463	256.0345	99.2171	366.0752	11.5	B
Northern elephant seal	24	13.7373	105.4984	3.6814	-13.2377	4.3	B
Northern elephant seal	24	47.2316	240.2812	23.5258	41.0192	0.2	B
Northern elephant seal	24	7.7646	297.4196	-3.5725	6.8923	12	B
Northern elephant seal	24	17.1618	258.3164	3.4942	16.8062	7.3	A
Northern elephant seal	24	4.6505	122.8085	2.5208	-3.9086	9.3	B
Northern elephant seal	24	0.4541	73.0653	-0.1322	-0.4344	0.4	A
Northern elephant seal	24	5.3506	80.9939	-0.8355	-5.2847	25.9	B
Northern elephant seal	24	0.8151	292.3263	-0.3096	0.754	18.7	A
Northern elephant seal	24	0.9992	352.6062	-0.9909	0.1286	22.2	B
Northern elephant seal	24	5.025	275.7272	-0.4996	4.9999	10.7	B
Northern elephant seal	24	1.7474	269.513	0.0151	1.7473	14.7	B
Northern elephant seal	24	1.4906	49.2922	-0.9721	-1.13	1.3	B
Northern elephant seal	24	4.7353	68.7914	-1.7116	-4.4145	20	B
Northern elephant seal	24	50.1193	255.783	12.4773	48.5845	17.5	B
Northern elephant seal	24	5.1125	241.8575	2.4128	4.5081	15.9	B
Northern elephant seal	24	190.3332	296.5929	-83.2051	170.2046	14.5	Z
Northern elephant seal	24	677.7864	102.3142	175.5127	-662.8319	1.3	B
Northern elephant seal	24	847.0051	71.5503	-226.3564	-804.3827	45.2	B
Northern elephant seal	25	0.4871	238.759	0.2526	0.4164	14.4	B
Northern elephant seal	25	2.1485	339.8142	-2.0165	0.7414	17.7	B
Northern elephant seal	25	99.5132	252.0378	31.2098	94.664	0.6	B
Northern elephant seal	25	1.4423	272.9407	-0.0739	1.4404	13.8	A
Northern elephant seal	25	35.7867	74.0442	-9.769	-34.4081	13.4	B
Northern elephant seal	25	9.3098	139.9966	7.1334	-5.9846	1.4	B
Northern elephant seal	25	7.6607	133.7774	5.3019	-5.5313	22.3	B
Northern elephant seal	25	3.6182	185.6357	3.6007	0.3553	10.7	B
Northern elephant seal	25	12.1518	71.2632	-3.8955	-11.5078	4.9	B
Northern elephant seal	25	1.2007	87.976	-0.0423	-1.2	0.8	B
Northern elephant seal	25	4.3854	235.0767	2.5114	3.5957	16.2	B
Northern elephant seal	25	3.6026	304.1173	-2.0201	2.9826	16.6	B
Northern elephant seal	25	6.8112	50.4548	-4.3348	-5.2523	19.1	B
Northern elephant seal	25	1.0956	135.1426	0.7766	-0.7727	0.6	B
Northern elephant seal	25	9.6312	238.8014	4.9938	8.2383	13.1	B
Northern elephant seal	25	2.0187	79.1938	-0.3782	-1.9829	0.5	B
Northern elephant seal	25	2.9038	244.6498	1.2437	2.6242	9.1	B
Northern elephant seal	25	4.2633	58.1647	-2.2479	-3.622	10.4	B
Northern elephant seal	25	6.5237	106.1694	1.8195	-6.2657	0.2	A
Northern elephant seal	25	10.3275	358.2498	-10.3227	0.3154	2.8	B
Northern elephant seal	25	14.5229	73.852	-4.0253	-13.9499	5.8	B
Northern elephant seal	25	25.8937	254.9047	6.7878	25.0003	17.7	B
Northern elephant seal	25	56.6606	75.9847	-13.5057	-54.9743	12.6	Z
Northern elephant seal	25	8.4496	232.0503	5.1995	6.6629	22.5	B
Northern elephant seal	25	1.5136	63.7284	-0.6698	-1.3572	14.1	B
Northern elephant seal	25	6.5293	255.7775	1.6071	6.3292	22.8	B
Northern elephant seal	25	296.996	294.1538	-116.2017	271.0315	10.2	B
Northern elephant seal	25	3.3756	330.599	-2.9406	1.6571	0.6	A
Northern elephant seal	25	29.2923	95.5815	2.9117	-29.1535	0.7	B
Northern elephant seal	25	7.0389	273.0287	-0.3682	7.029	13.8	B
Northern elephant seal	25	2.0023	334.5476	-1.8079	0.8605	0.8	B

Northern elephant seal	25	3.6984	289.0704	-1.2074	3.4954	1.2	A
Northern elephant seal	25	14.4617	134.0298	10.0595	-10.3976	9.1	B
Northern elephant seal	25	1.3328	217.9179	1.0515	0.819	1	B
Northern elephant seal	25	2.571	77.6083	-0.5512	-2.5111	3.2	B
Northern elephant seal	25	2.2079	104.2677	0.5445	-2.1398	22.5	B
Northern elephant seal	25	2.5948	93.9759	0.1804	-2.5885	15.3	B
Northern elephant seal	25	17.5526	23.7723	-16.0595	-7.0755	6.9	B
Northern elephant seal	25	34.5534	289.6781	-11.5537	32.5355	0.5	B
Northern elephant seal	25	1.2699	88.555	-0.0319	-1.2695	18.8	B
Northern elephant seal	25	1.7482	216.02	1.4141	1.0281	21.5	B
Northern elephant seal	25	2.4423	334.7302	-2.2085	1.0426	0.8	A
Northern elephant seal	25	9.4387	117.6353	4.3834	-8.3619	14.7	B
Northern elephant seal	25	4.2892	106.9035	1.2484	-4.1039	32	B
Northern elephant seal	25	2.3318	77.2455	-0.5144	-2.2743	0.2	0
Northern elephant seal	25	1.6389	57.5693	-0.8788	-1.3833	6.3	A
Northern elephant seal	25	1.4348	87.4109	-0.0647	-1.4334	9.6	B
Northern elephant seal	25	0.8194	277.9674	-0.1135	0.8114	0.2	A
Northern elephant seal	25	1.2681	240.8548	0.6177	1.1075	1.2	A
Northern elephant seal	25	14.0695	279.2212	-2.2398	13.8877	2.6	B
Northern elephant seal	25	29.658	243.6623	13.2129	26.5794	6.3	B
Northern elephant seal	25	1.3632	311.3273	-0.9001	1.0237	9.3	B
Northern elephant seal	25	1.3647	120.5171	0.6931	-1.1756	0.2	B
Northern elephant seal	25	0.14	145.1032	0.1148	-0.0801	2.8	B
Northern elephant seal	25	23.6371	96.0959	2.5528	-23.5034	19.3	B
Northern elephant seal	25	1.444	245.1206	0.6076	1.31	2	B
Northern elephant seal	25	0.4967	187.1675	0.4928	0.062	22.7	B
Northern elephant seal	25	3.0449	82.669	-0.3879	-3.02	14.4	1
Northern elephant seal	25	1.7086	235.0064	0.98	1.3997	0.1	B
Northern elephant seal	25	8.8331	118.2794	4.1891	-7.7788	8.1	B
Northern elephant seal	25	4.9737	251.5033	1.5795	4.7168	13.3	B
Northern elephant seal	25	3.085	296.4141	-1.3718	2.7629	47.4	B
Northern elephant seal	25	7.1916	266.5462	0.4367	7.1786	9.6	B
Northern elephant seal	25	5.2686	235.7107	2.9693	4.3529	3.9	B
Northern elephant seal	25	18.5973	60.3348	-9.1886	-16.1598	6.2	B
Northern elephant seal	25	10.563	303.8325	-5.8765	8.7743	4.5	B
Northern elephant seal	25	4.1021	65.4065	-1.7064	-3.73	0.8	B
Northern elephant seal	25	1717.8332	97.8937	419.6156	-1710.2137	15	B
Northern elephant seal	25	849.9141	101.7648	214.881	-832.9406	16.9	B
Northern elephant seal	25	5.3433	293.3587	-2.1172	4.9054	0.7	B
Northern elephant seal	25	27.1407	271.7737	-0.7977	27.1277	0.7	B
Northern elephant seal	25	28.8232	103.7241	6.8833	-28.0003	0.2	B
Northern elephant seal	25	9.6263	117.3742	4.4304	-8.5483	13.3	B
Northern elephant seal	25	4.0058	188.9722	3.9568	0.6247	12.3	B
Northern elephant seal	25	9.2021	201.2729	8.5757	3.3386	0.6	B
Northern elephant seal	25	7.2825	42.8247	-5.3399	-4.9503	29.1	B
Northern elephant seal	25	13.8043	74.473	-3.6852	-13.3005	9.4	B
Northern elephant seal	25	11.9188	291.2583	-4.3144	11.1078	1	B
Northern elephant seal	25	6.5936	39.8133	-5.0638	-4.2218	0.7	B
Northern elephant seal	25	2.9484	280.5622	-0.54	2.8984	4.5	A
Northern elephant seal	25	8.7761	125.8923	5.148	-7.1097	10.4	B
Northern elephant seal	25	28.0847	104.165	6.9165	-27.2308	4	B
Northern elephant seal	25	1.257	193.3099	1.2233	0.2894	0.9	0
Northern elephant seal	26	10.4972	156.0949	9.5978	-4.2537	20	B
Northern elephant seal	26	15.1457	226.8613	10.3633	11.0518	1.8	B
Northern elephant seal	26	23.2053	102.3021	4.9746	-22.6725	24.3	B
Northern elephant seal	26	7.8603	69.4074	-2.7614	-7.358	18.4	B
Northern elephant seal	26	19.5744	71.1206	-6.3136	-18.5213	3.6	B
Northern elephant seal	26	18.0121	312.2002	-12.0864	13.3434	7.8	A
Northern elephant seal	26	8.2679	96.6146	0.9573	-8.2129	0.4	B
Northern elephant seal	26	856.7449	288.6341	-225.4303	813.1005	6.8	A
Northern elephant seal	26	1335.5327	260.7243	352.7003	1324.5432	13.9	B
Northern elephant seal	26	1128.1429	258.8814	314.9498	1110.7946	8.1	A
Northern elephant seal	26	25.42	247.7143	9.6825	23.5212	23.1	B
Northern elephant seal	26	7.5053	70.4031	-2.5134	-7.0705	11.3	B
Northern elephant seal	26	0.7785	146.1231	0.6464	-0.434	18.5	A
Northern elephant seal	26	5.1898	306.5609	-3.0901	4.1686	15.2	B
Northern elephant seal	26	1.6794	15.8611	-1.6155	-0.459	9.3	B
Northern elephant seal	26	2.852	306.8214	-1.7088	2.283	14.6	B

Northern elephant seal	26	1.0288	182.3481	1.0279	0.0421	14.4	B
Northern elephant seal	26	0.8371	121.1211	0.4327	-0.7166	31	A
Northern elephant seal	26	24.7811	250.2478	8.419	23.3231	0.5	A
Northern elephant seal	26	9.1967	45.0123	-6.4982	-6.5044	19.6	B
Northern elephant seal	26	2.5734	243.7689	1.1378	2.3084	26.9	B
Northern elephant seal	26	3.4407	216.8149	2.7549	2.0618	1	A
Northern elephant seal	26	3.631	113.572	1.4529	-3.328	15.9	B
Northern elephant seal	26	5.0911	98.5056	0.7551	-5.0351	1	B
Northern elephant seal	26	9.7698	261.7842	1.4037	9.6696	0.2	A
Northern elephant seal	26	2.5853	178.5084	2.5844	-0.0673	18.8	B
Northern elephant seal	26	15.2397	255.0666	3.9445	14.725	1	B
Northern elephant seal	26	4.2654	153.5129	3.8179	-1.9023	19.7	B
Northern elephant seal	26	2.8725	65.0691	-1.2103	-2.6049	23.1	B
Northern elephant seal	26	13.3877	101.3374	2.645	-13.1265	15	B
Northern elephant seal	26	9.2071	290.6659	-3.2437	8.6146	0.4	B
Northern elephant seal	26	5.2457	98.1297	0.7439	-5.1929	0.7	B
Northern elephant seal	26	13.9442	68.8064	-5.0285	-13.0011	0.4	B
Northern elephant seal	26	8.3469	29.3689	-7.273	-4.0936	4	B
Northern elephant seal	26	3.4646	239.2219	1.7735	2.9767	15.7	B
Northern elephant seal	26	9.9707	307.1589	-6.0183	7.9463	18.1	B
Northern elephant seal	26	0.7153	6.6115	-0.7106	-0.0824	0.5	0
Northern elephant seal	26	19.284	67.0635	-7.4948	-17.7593	9.9	A
Northern elephant seal	26	9.6427	115.5448	4.1629	-8.7001	17.5	B
Northern elephant seal	26	11.1642	313.322	-7.6556	8.1221	18.3	B
Northern elephant seal	26	6.726	357.2981	-6.7185	0.3171	1.2	B
Northern elephant seal	27	9.4946	83.7694	-1.0251	-9.4385	7.6	B
Northern elephant seal	27	16.9048	90.0531	0.0328	-16.9048	12.4	B
Northern elephant seal	27	2.0249	297.346	-0.93	1.7986	11.7	B
Northern elephant seal	27	17.315	98.8224	2.6733	-17.1101	1.6	B
Northern elephant seal	27	857.6613	287.7075	-219.1784	817.9284	19.5	A
Northern elephant seal	27	2.9023	281.3113	-0.5687	2.8459	0.4	B
Northern elephant seal	27	127.5979	285.0338	-31.9019	123.2358	15.5	B
Northern elephant seal	27	1.8802	22.2044	-1.7408	-0.7106	8.2	B
Northern elephant seal	27	3.8225	38.5403	-2.9894	-2.3816	0.7	B
Northern elephant seal	27	350.1967	256.5174	91.0183	340.6629	7.5	B
Northern elephant seal	27	82.7247	78.9476	-15.3339	-81.1919	17.4	B
Northern elephant seal	27	6.6704	118.184	3.153	-5.8795	0.3	B
Northern elephant seal	27	6.0247	105.6801	1.6303	-5.8005	0.2	B
Northern elephant seal	27	2.215	301.76	-1.1657	1.8833	7.4	B
Northern elephant seal	39	23.1445	257.5416	5.0253	22.5995	0	A
Northern elephant seal	39	33.7954	260.0933	5.8846	33.2916	0	Z
Northern elephant seal	39	2.2167	116.2559	0.9809	-1.988	0	B
Northern elephant seal	39	10.5213	70.5782	-3.4921	-9.9226	0	A
Northern elephant seal	39	29.2547	272.3384	-1.1355	29.2304	0	B
Northern elephant seal	39	137.4189	249.578	49.0883	128.7856	0	B
Northern elephant seal	39	25.389	207.1446	22.6018	11.5834	0	A
Northern elephant seal	39	35.0785	268.8801	0.7727	35.0719	0	B
Northern elephant seal	39	0.1881	89.8829	-0.0004	-0.1881	0	B
Northern elephant seal	39	2.2109	291.6894	-0.8168	2.0544	0	B
Northern elephant seal	39	5.2988	84.5478	-0.5014	-5.2748	0	A
Northern elephant seal	39	7.5715	246.9458	2.9686	6.9668	0	A
Northern elephant seal	39	8.5555	70.6244	-2.8336	-8.0709	0	B
Northern elephant seal	39	6.1852	252.0975	1.9039	5.8857	0	A
Northern elephant seal	39	69.3056	74.7274	-17.9285	-66.8587	0	Z
Northern elephant seal	39	25.2844	305.7504	-14.7413	20.52	0	Z
Northern elephant seal	39	3.5182	265.8619	0.2548	3.509	0	B
Northern elephant seal	39	1.7686	127.9485	1.0877	-1.3946	0	A
Northern elephant seal	39	4.5203	327.3935	-3.8074	2.4358	0	A
Northern elephant seal	39	3.5984	108.9594	1.17	-3.4032	0	B
Northern elephant seal	39	7.1373	243.175	3.2239	6.3693	0	B
Northern elephant seal	39	3.0928	226.5491	2.1274	2.2453	0	A
Northern elephant seal	39	78.5204	297.7267	-36.1751	69.5049	0	B
Northern elephant seal	39	141.7093	241.0097	69.8306	123.9563	0	B
Northern elephant seal	39	19.0401	293.0185	-7.4224	17.5241	0	B
Northern elephant seal	39	4.1822	96.9326	0.5061	-4.1516	0	0
Northern elephant seal	39	31.5221	110.1937	10.9462	-29.5845	0	B
Northern elephant seal	39	18.7218	295.6184	-8.0738	16.8814	0	B
Northern elephant seal	39	2.2769	169.7842	2.2409	-0.4038	0	A

Northern elephant seal	39	3.9575	68.7307	-1.4346	-3.6879	0	A
Northern elephant seal	39	4.8754	323.6214	-3.9247	2.8917	0	A
Northern elephant seal	39	3.1599	323.8604	-2.5516	1.8636	0	A
Northern elephant seal	39	86.7317	133.0651	59.4925	-63.3639	0	B
Northern elephant seal	39	18.1952	116.9425	8.2617	-16.2203	0	0
Northern elephant seal	39	0.4575	330.4977	-0.3982	0.2253	0	2
Northern elephant seal	39	7.2413	159.1858	6.7691	-2.5731	0	A
Northern elephant seal	39	3.2906	122.7314	1.7798	-2.7681	0	B
Northern elephant seal	39	28.1872	208.7075	24.7338	13.5394	0	B
Northern elephant seal	39	6.0092	173.5311	5.971	-0.677	0	B
